# Brain profiling in murine colitis and human epilepsy reveals neutrophils and TNFα as mediators of neuronal hyperexcitability

**DOI:** 10.1186/s12974-021-02262-4

**Published:** 2021-09-12

**Authors:** Sarah E. Barnes, Kristy A. Zera, Geoffrey T. Ivison, Marion S. Buckwalter, Edgar G. Engleman

**Affiliations:** 1 Department of Pathology, Stanford University, Stanford, CA USA; 2 Department of Neurology, Stanford University, Stanford, CA USA; 3 Department of Infectious Diseases, Stanford University, Stanford, CA USA

**Keywords:** Colitis, Inflammatory bowel disease, Epilepsy, Neuronal hyperexcitability, Neutrophils, TNFα

## Abstract

**Background:**

Patients with chronic inflammatory disorders such as inflammatory bowel disease frequently experience neurological complications including epilepsy, depression, attention deficit disorders, migraines, and dementia. However, the mechanistic basis for these associations is unknown. Given that many patients are unresponsive to existing medications or experience debilitating side effects, novel therapeutics that target the underlying pathophysiology of these conditions are urgently needed.

**Methods:**

Because intestinal disorders such as inflammatory bowel disease are robustly associated with neurological symptoms, we used three different mouse models of colitis to investigate the impact of peripheral inflammatory disease on the brain. We assessed neuronal hyperexcitability, which is associated with many neurological symptoms, by measuring seizure threshold in healthy and colitic mice. We profiled the neuroinflammatory phenotype of colitic mice and used depletion and neutralization assays to identify the specific mediators responsible for colitis-induced neuronal hyperexcitability. To determine whether our findings in murine models overlapped with a human phenotype, we performed gene expression profiling, pathway analysis, and deconvolution on microarray data from hyperexcitable human brain tissue from patients with epilepsy.

**Results:**

We observed that murine colitis induces neuroinflammation characterized by increased pro-inflammatory cytokine production, decreased tight junction protein expression, and infiltration of monocytes and neutrophils into the brain. We also observed sustained neuronal hyperexcitability in colitic mice. Colitis-induced neuronal hyperexcitability was ameliorated by neutrophil depletion or TNFα blockade. Gene expression profiling of hyperexcitable brain tissue resected from patients with epilepsy also revealed a remarkably similar pathology to that seen in the brains of colitic mice, including neutrophil infiltration and high TNFα expression.

**Conclusions:**

Our results reveal neutrophils and TNFα as central regulators of neuronal hyperexcitability of diverse etiology. Thus, there is a strong rationale for evaluating anti-inflammatory agents, including clinically approved TNFα inhibitors, for the treatment of neurological and psychiatric symptoms present in, and potentially independent of, a diagnosed inflammatory disorder.

**Supplementary Information:**

The online version contains supplementary material available at 10.1186/s12974-021-02262-4.

## Background

Patients with chronic inflammatory conditions frequently experience neurological and psychiatric complications including epilepsy, depression, attention deficit and hyperactivity disorders, migraines, and dementia [1–7]. A meta-analysis of data from >25 million individuals revealed associations between several autoinflammatory conditions and psychiatric disorders [1], and a retrospective population-level study found that the risk of epilepsy was increased almost fourfold in patients diagnosed with an autoimmune disorder [8]. Our lack of understanding of the mechanisms underlying these disorders and their neuropsychiatric complications makes it very difficult to identify which drugs will be most helpful for individual patients. Furthermore, psychiatric and neurological medications frequently produce disruptive and debilitating side effects such as cognitive impairment, sedation, exacerbation of other neurological and psychiatric symptoms, and sexual dysfunction [9, 10]. These severe side effects combined with the large number of patients who do not respond to existing therapies provide a strong rationale to examine the mechanistic connection between peripheral inflammation and the development of neurological symptoms. Given that 4.5% of Americans suffer from an autoinflammatory disease [11], strategies to protect this population from developing neurological complications, as well as treat them, are urgently needed.

There are many hypotheses as to why peripheral inflammatory disorders and neurological conditions co-occur; however, there have been few mechanistic studies demonstrating a causal relationship, and investigations of these mechanisms have yielded contradictory results [12–18]. Other studies have utilized models of inflammation induced by large doses of nonspecific stimuli such as lipopolysaccharide (LPS), which do not closely mimic human disease [19–24]. Furthermore, neuroinflammation can exist in patients with neurological disorders who do not have a diagnosed autoinflammatory disease [25–29]. Because the vast majority of studies investigating the occurrence and impact of neuroinflammation on brain function have utilized models of primary neurological disease, it is difficult to uncouple the role of the primary neurological pathology from that of inflammation. To more clearly understand the mechanisms by which peripheral inflammatory disease causes neurological symptoms, it is therefore necessary to employ models of peripheral inflammation in which the brain is not initially involved.

Because intestinal disorders such as inflammatory bowel disease (IBD) are robustly associated with neurological symptoms [2, 4, 8, 30–42], we used three different mouse models of colitis to investigate the impact of peripheral inflammatory disease on the brain. We focused on measurements of neuronal excitability, since many neurological and psychiatric symptoms are associated with neuronal hyperexcitability.

Our experiments show that neuroinflammation and neuronal hyperexcitability develop in all three colitis models as a consequence of TNFα-secreting neutrophils that infiltrate the brain. Moreover, we corroborated our findings in resected brain tissue from patients with epilepsy who had no known peripheral inflammatory disease, suggesting that TNFα-mediated neuroinflammation contributes to neuronal hyperexcitability of diverse etiology.

## Methods

### Animals

C57BL/6J, *Rag2*^−/−^, and CCR2^RFP/RFP^ mice were obtained from The Jackson Laboratory. *Rag2*^−/−^ and CCR2^RFP/RFP^ mice were then bred in-house at the Stanford Blood Center. Mice were age- and sex-matched for experiments. Experiments were conducted on mice between the ages of 6–8 weeks.

### Seizure threshold measurements

Individual mice were placed in an airtight chamber. Using a syringe pump, 10% flurothyl (Sigma Aldrich, #287571) in ethanol was administered at a rate of 100μL/min onto a filter paper placed inside of the chamber, causing the flurothyl to evaporate within the chamber. Latency to both seizure onset and seizure generalization were measured. Seizure onset was noted as the first myoclonic jerk. Seizure generalization was noted as the first loss of posture.

### Colitis

We utilized three murine colitis models. For acute DSS-induced colitis, mice were given 3% DSS (Hardman Chemicals, or 3.5% from TdB Labs) in drinking water for 6 days, followed by 9 days of regular drinking water. For chronic DSS-induced colitis, this cycle was repeated two more times with an additional 7-day break between cycles. For TCT-induced colitis, CD4^+^ T cells were enriched from C57BL/6J spleens. Enriched cells were then FACS-sorted to obtain naïve CD4^+^ T cells (CD45^+^CD4^+^CD45RB^hi^CD25^lo^) and Tregs (CD45^+^CD4^+^CD45RB^lo^CD25^hi^). Experimental *Rag2*^−/−^ mice received 500,000 naïve CD4^+^ T cells, and control mice received 500,000 naïve CD4^+^ T cells in addition to 100,000 Tregs.

### Murine tissue harvesting and processing

Mice were anesthetized using isoflurane and then perfused with a minimum of 10mL PBS, until the liver was fully perfused. Brains were harvested and excluded if perfusion was incomplete. A GentleMACS dissociator was used to homogenize brains in complete RPMI containing 1mg/mL collagenase IV (Worthington Biochemical, #LS004188). Brains were further digested by shaking inside of a 37°C incubator for 30 min. The tissue was passed through a 70-μm filter and washed in complete RPMI. Cells were separated using a 30% Percoll (GE Healthcare, #17089101) gradient and washed again in complete RPMI.

### Flow cytometry

Single-cell suspensions were generated from harvested tissue. Cells were washed in PBS and then stained with 1:1000 Fc blocker and Live/Dead Aqua in PBS for 10 min at 4°C. At this point, cells were either stained or fixed in 2% paraformaldehyde for 10 min at 4°C and stained the following day. For intracellular stains, cells were fixed and permeabilized using the Foxp3/Transcription Factor Staining Buffer Kit (eBioscience) prior to staining. Cells were washed in FACS buffer (PBS + 10% FBS + 1mM EDTA) and stained using fluorescently labeled antibodies for 20 min at 4°C. Cells were analyzed using a BD LSR Fortessa or BD FACS Canto.

### In vivo labeling of border-associated leukocytes

Mice were anesthetized using isoflurane and then retro-orbitally injected with 7.5μg of fluorescently labeled α-CD45 antibody in PBS. Three minutes later, mice were immediately perfused with a minimum of 10mL PBS, until the liver was fully perfused. Single-cell suspensions were generated from harvested brains and used for flow cytometry, and tissue sections were obtained for immunofluorescence. Cells were stained ex vivo with an α-CD45 antibody attached to a different fluorophore. Cells that were labeled with both antibodies were deemed border-associated, and cells that were labeled only with the ex vivo-administered antibody were deemed parenchymal.

### Protein and transcript quantification

Plasma was obtained by collecting blood retro-orbitally with heparin capillary tubes into plasma microtainer tubes. Tubes were spun at 6000rpm for 6 min. Plasma cytokine levels were measured using a cytokine bead array (BD Biosciences). To measure transcript levels, RNA was obtained from perfused, flash-frozen brains. Fast SYBR Green PCR mix (Applied Biosystems) and the 7900HT real-time PCR instrument were used to conduct qRT-PCR.

### Immunohistochemistry and H&E staining

Mice were sedated using isoflurane and then perfused with PBS. Brains were collected and drop-fixed in 2% paraformaldehyde overnight at 4°C. They were preserved in 30% sucrose at 4°C. A freezing microtome (Microm HM430) was used to collect 40-μm sections, which were stored in a cryoprotectant medium (30% glycerin, 30% ethylene glycol, 40% 0.5M sodium phosphate buffer) at 20°C. A standard immunohistochemistry protocol was performed to stain free-floating sections. Sections were blocked with 3% rabbit serum for 1 h. The tissue was then incubated at 4°C overnight in primary α-CD68 antibody (rat, 1:1000, BioRad MCA1957S). Tissue was incubated for 1 h in secondary rabbit *a*-rat IgG antibody (1:500, Vector Laboratories, #BA-4001). Tissue was then treated with Avidin-Biotin Complex solution (Vector Laboratories, #PK-6100) for 1 h and treated for 5 min with filtered DAB solution (Sigma Aldrich, #D5905). Sections were mounted onto glass slides, air-dried overnight, and coverslipped with Entellan (Electron Microscopy Sciences 14,800). Quantification of area stained by CD68 was performed on 2 sections per brain for the cortex and 1 section per brain for the hippocampus by a researcher blinded to treatment groups using ImageJ software (National Institutes of Health). For in vivo labeling of leukocytes, biotinylated α-CD45 (7.5μg, BioLegend #103104) was administered in vivo with a secondary streptavidin-488 stain on tissue sections (1:200, Thermo Fisher #32354) and a pre-conjugated CD45-PE antibody (1:500, BD Biosciences #561087) was used for ex vivo staining. For H&E staining, brains were harvested as previously described, fixed in 2% paraformaldehyde overnight, and then stored in 70% ethanol. H&E staining was performed by the Stanford Pathology Department Histology Services Core. For both IHC and H&E, slides were imaged using a Keyence BZ-X700 microscope.

### Cellular depletion and cytokine neutralization

Neutrophils were depleted by injecting mice intraperitoneally once daily with 500μg α-Ly6G antibody (clone 1A8, Bio X Cell) from days 6 to 14 after the onset of DSS treatment. Control mice received 500μg isotype control IgG2A (clone 2A8, Bio X Cell) according to the same treatment schedule. Monocytes were depleted by injecting mice retro-orbitally once daily with 200μL clodronate liposomes (Clodrosome) from days 6 to 14 after the onset of DSS treatment. Control mice received 200μL of PBS liposomes (Clodrosome) according to the same treatment schedule. TNFα was neutralized by injecting mice intraperitoneally once daily with 500μg α-TNFα antibody (clone XT3.11, Bio X Cell) from days 6 to 14 after the onset of DSS treatment. Control mice received 500μg isotype control IgG1 (clone HRPN, Bio X Cell). Treatment schedules were determined using longitudinal analysis of immune cell frequencies in the peripheral blood of colitic mice.

### Deconvolution, gene expression, and pathway analysis

Agilent feature extraction files were processed using the Significance Analysis of Microarrays (SAM) R package [43]. Deconvolution was performed using CIBERSORTx [44]. PCA was performed using the Scikit-Learn PCA package for Python [45]. Differentially regulated genes were identified as having *p*-value < 0.05 and log2(fold change) > 1. Gene ontology analysis was performed using the Gene Ontology Resource from the GO Consortium. Pathway analysis was performed using Enrichr [46, 47], and network analysis was performed using Cytoscape [48]. A *p*-value < 0.05 was used to determine significant overlap between upregulated genes and pre-defined gene sets from the Kyoto Encyclopedia of Genes and Genomes (KEGG).

### Statistical analysis

Unless otherwise specified, experimental data were analyzed using the Mann-Whitney *U* test. For seizure threshold experiments, measurements were acquired and pooled over 3–4 days. Two-way ANOVAs were used to identify differences in measurement day as well as experimental treatment. Simple linear regressions were calculated to determine goodness of fit and statistically significant slopes. Longitudinal body weight measurements were compared using the Holm-Sidak method for performing multiple *t*-tests. Results are reported as mean with individual data points or mean ± SEM: *p* < 0.05 = *; *p* < 0.01 = **; *p* < 0.001 = ***; *p* < 0.0001 = ****. All statistics were calculated using Prism (GraphPad Software).

## Results

### Acute and chronic colitis cause a sustained reduction in seizure threshold

**Fig. 1:**
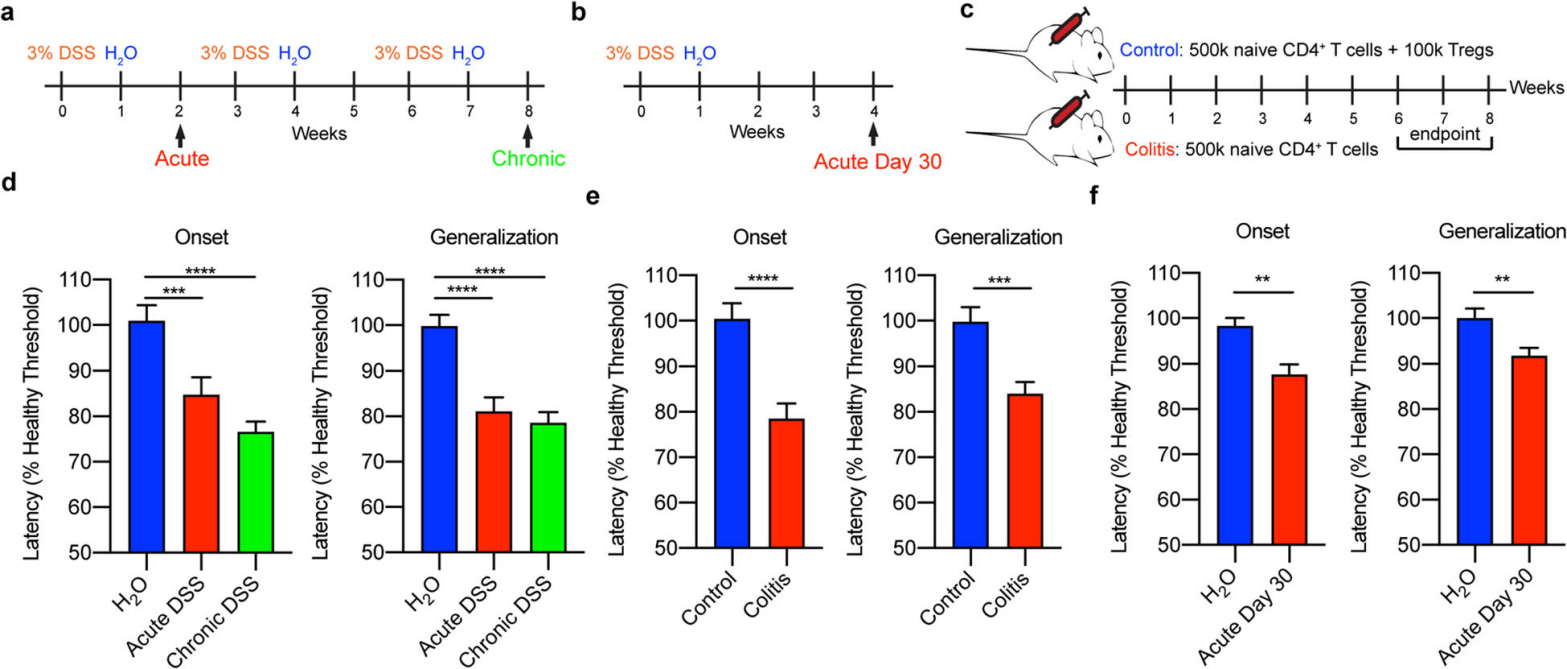
Acute and chronic colitis cause a sustained reduction in seizure threshold. Experimental protocols for **a** acute and chronic DSS-induced colitis, **b** 30 days following acute DSS-induced colitis, and **c** TCT-induced colitis. **d**–**f** Latencies to seizure onset (left) and generalization (right) following flurothyl administration in mice with acute or chronic DSS-induced colitis (**d**), mice with TCT-induced colitis (**e**), and mice 30 days following initiation of acute DSS treatment (**f**). Data represent the combined analysis of three experiments containing 5–10 mice/condition/experiment. **p < 0.01, ***p < 0.001, ****p < 0.0001

Our studies utilized three mouse colitis models, each of which is known to recapitulate various aspects of IBD: acute dextran sodium sulfate (DSS)-induced colitis (Fig. 1a, b), chronic DSS-induced colitis (Fig. 1a), and T cell transfer (TCT)-induced colitis (Fig. 1c). DSS impairs epithelial barrier function within the gut, resulting in the entry of intestinal microorganisms into the lamina propria and subsequent induction of innate and adaptive immune responses [49]. Although DSS has not been shown to enter the brain [50], we also utilized the TCT-induced colitis model to confirm that changes in seizure threshold were due to inflammation rather than any direct effects of DSS on the brain. In this model, regulatory T cell (Treg)-depleted naïve CD4^+^ T cells are retro-orbitally transferred into *Rag2*^*−/−*^ mice, which lack B and T cells; control mice receive naïve CD4^+^ T cells in addition to Tregs. In the absence of Tregs, naïve CD4^+^ T cells mount an immune response targeting the intestinal microbiota, resembling that of patients with IBD [49].

To measure neuronal hyperexcitability in each of the IBD models, we measured seizure threshold in response to flurothyl, a γ-aminobutyric acid (GABA) receptor antagonist. The dosage of flurothyl required to induce a seizure is inversely proportional to neuronal excitability; mice with hyperexcitability display reduced seizure thresholds [51]. Flurothyl was chosen for these experiments for three reasons: (1) it is administered via inhalation, which allows for control of dosing and limits mortality; (2) it is not metabolized and has no known direct influences on the immune response; and (3) unlike transgenic spontaneous seizure models, confounding effects of genetic mutations on immune responses and seizure-induced inflammation are avoided. We measured both the time to the first myoclonic jerk (latency to seizure onset) and the time to loss of posture (latency to seizure generalization), indicating that the seizure has spread widely throughout the brain. Regardless of the modality of colitis induction, mice with colitis displayed reduced latency to seizure onset and generalization compared to healthy mice (Fig. 1d, e). Notably, seizure onset and generalization continued to be more rapid in mice with acute colitis even after 30 days following the onset of acute colitis (Fig. 1f), indicating a long-lasting neuroexcitatory phenotype following acute DSS.

### Colitis induces monocyte and neutrophil infiltration of the brain, increased expression of neuroinflammatory cytokines, and decreased expression of tight junction proteins, but minimal microglial activation

**Fig. 2:**
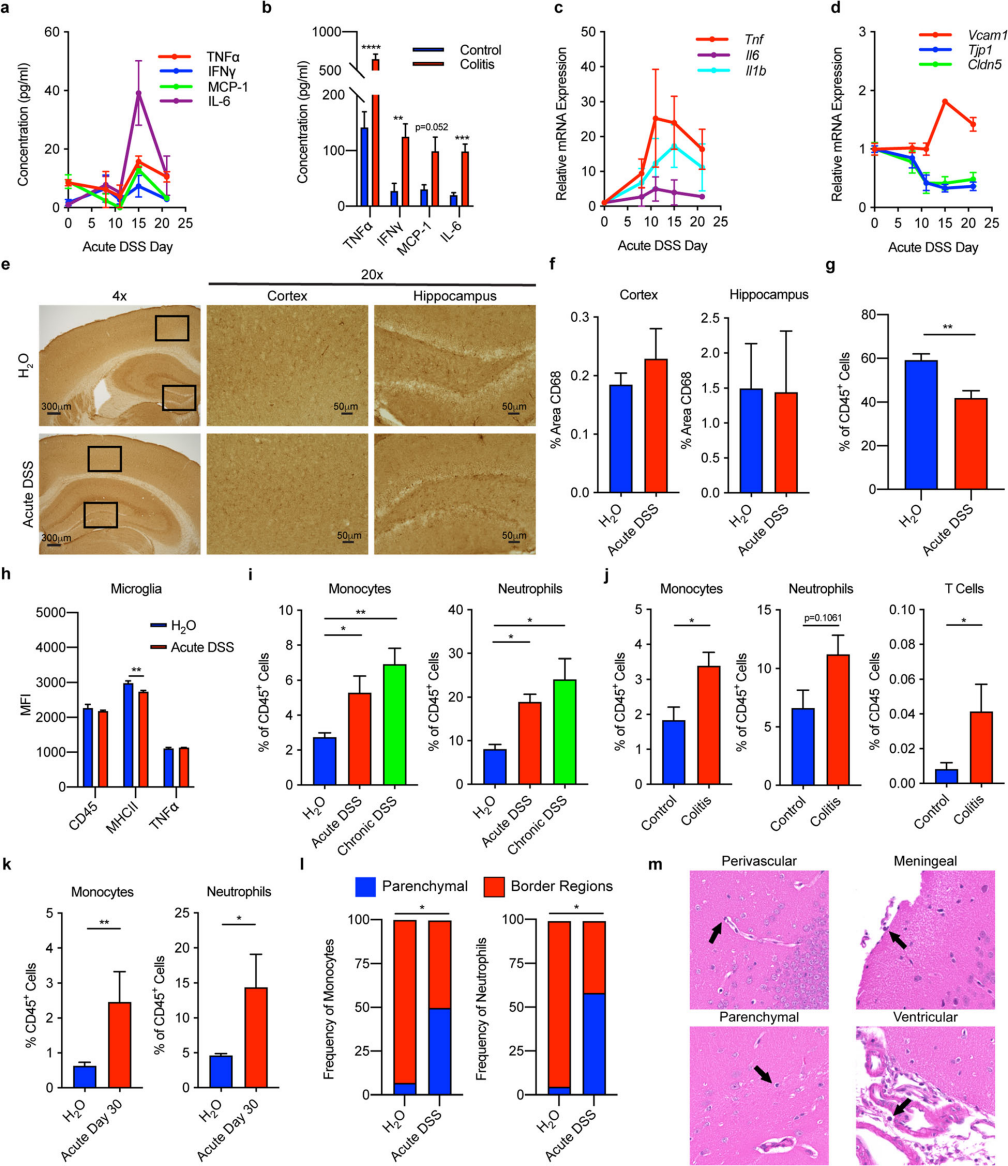
Colitis induces neuroinflammation with monocyte and neutrophil infiltration and increased cytokine production. **a**, **b** Plasma cytokine concentrations in mice with acute DSS-induced colitis (**a**) and TCT-induced colitis (**b**). **c**, **d** Transcription of cytokines (**c**), and *Vcam1*, *Tjp1*, and *Cldn5* (**d**) in the brains of mice with acute DSS-induced colitis. **e**, **f** Representative immunohistochemistry stains (**e**) and quantification (**f**) of CD68 in hippocampal and cortex sections from mice with acute DSS-induced colitis. **g**, **h** Flow cytometric analysis of microglia (CD45^lo^CX3CR1^hi^CD11b^+^) showing their frequency (**g**) and phenotype (**h**) from mice with acute DSS-induced colitis. **i** Flow cytometric quantification of monocytes (CD45^+^CD11b^+^Ly6G^−^Ly6C^+^MHCII^−^) and neutrophils (CD45^+^CD11b^+^Ly6G^+^) in mice with acute and chronic DSS-induced colitis. **j** Flow cytometric quantification of monocytes, neutrophils, and T cells (CD45^+^CD11b^-^CD3^+^) in mice with TCT-induced colitis. **k** Frequencies of monocytes and neutrophils 30 days following initiation of acute DSS-induced colitis. **l** Distribution of monocytes and neutrophils in the brains of mice with acute DSS-induced colitis. Single-cell suspensions of harvested tissue were analyzed by flow cytometry. **m** H&E stain of brain sections from mice with acute DSS-induced colitis. *n* = 5–10 mice/group, representative of 3–5 replicate experiments. *p < 0.05, **p < 0.01, ***p < 0.001, ****p < 0.0001

In support of the possibility that colitis-associated inflammatory factors contribute to neuroinflammation, we observed increased plasma concentrations of TNFα, MCP-1/CCL2, IL-6, and IFNγ in colitic mice (Fig. 2a, b), as well as increased transcription of *Tnf*, *Il1b*, and *Il6* in their brains (Fig. 2c). We also observed increased transcription of the adhesion molecule VCAM-1 (*Vcam1*), a marker of vascular neuroinflammation, and decreased transcription of the tight junction proteins ZO-1 (*Tjp1*) and claudin-5 (*Cldn5*) in the brains of colitic mice, indicative of blood-brain barrier (BBB) disruption (Fig. 2d). Transcription of *Tnf*, *Il1b*, and *Il6* each correlated with decreased transcription of *Tjp1*, but not *Cldn5*, suggesting that these cytokines may reduce BBB integrity via ZO-1 downregulation (Supplemental Figure 1). Transcription of *Tnf*, *Il1b*, *Il6*, and *Vcam1* all remained elevated, and *Tjp1* and *Cldn5* remained decreased, for 21 days following initiation of DSS treatment (beyond the point at which plasma cytokine concentrations had returned to baseline), further indicating that acute colitis induces a sustained neuroinflammatory response.

Because microglia are known to play a central role in neuroinflammation [22, 24, 52–56], we characterized these brain-resident myeloid cells using immunohistochemistry and flow cytometry. However, we found no evidence of microglia activation in colitic mice, as reflected by unchanged CD68 expression in the cortex and hippocampus (Fig. 2e, f), reduced microglia frequency (likely attributable to infiltrating cells, Fig. 2g), and no increases in microglial CD45, TNFα, or MHCII expression (Fig. 2h). Based on the increased transcription of the vascular adhesion molecule VCAM-1 in the brains of colitic mice (Fig. 2d), we hypothesized that peripheral immune cells adhered to brain vasculature and/or infiltrated the brains of colitic mice. Flow cytometric analysis revealed increased frequencies of monocytes and neutrophils in the brains of colitic mice (Fig. 2i, j), but no meaningful changes in the frequencies of other immune cells (Supplemental Figure 2). Frequencies of monocytes and neutrophils remained elevated 30 days following the onset of acute colitis, further indicating that acute colitis induced a sustained neuroinflammatory response (Fig. 2k). We observed a small increase in T cell frequency in mice with TCT-, but not DSS-, induced colitis (Fig. 2j). Despite this increase, T cells were <1% of total leukocytes, suggesting minimal involvement of these cells in the colitis-induced neuroinflammatory process.

To determine the location of these cells in the brain, we retro-orbitally injected mice with a fluorescently labeled α-CD45 antibody; cells in the border regions (vasculature, perivascular spaces, ventricles, and meninges), but not the parenchyma, were labeled with the α-CD45 antibody [55]. Using flow cytometry, we observed that almost all the monocytes and neutrophils in the brains of healthy control mice stained positive with the α-CD45 antibody, indicating that they were in border regions (Fig. 2l, Supplemental Figure 4). By contrast, roughly half of the monocytes and neutrophils present in the brains of colitic mice did not stain with the α-CD45 antibody, indicating that they were located in the parenchyma. Using an H&E stain, we observed leukocytes in both the parenchyma—which does not normally contain leukocytes—and border regions of the hippocampus and cortex in colitic mice (Fig. 2m).

### Neutrophils, but not monocytes, contribute to neuronal hyperexcitability following acute colitis via TNFα

**Fig. 3:**
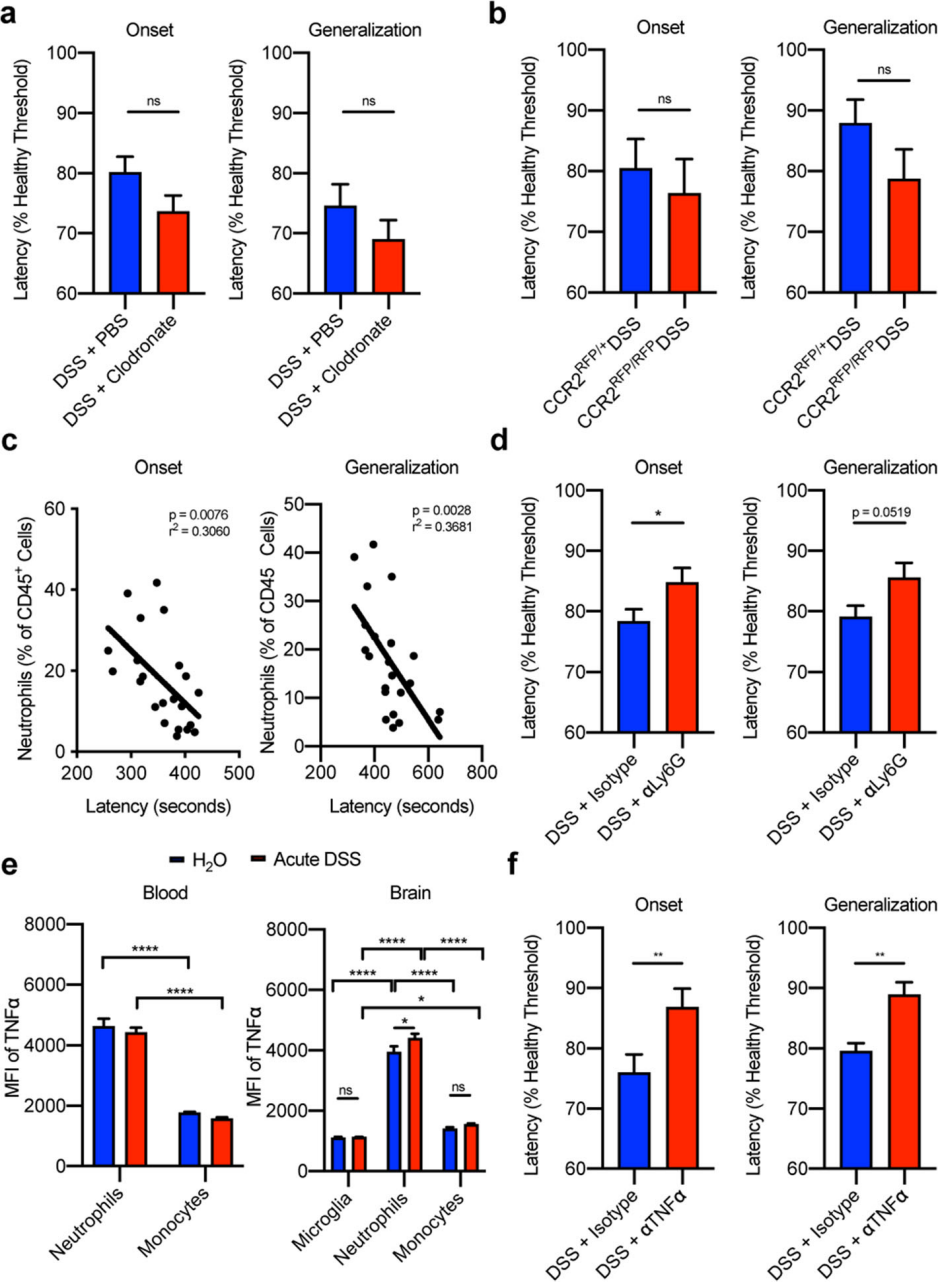
Neutrophils, but not monocytes, contribute to neuronal hyperexcitability following acute colitis via TNFα. **a**, **b** Latencies to seizure onset (left) and generalization (right) in clodronate-treated mice (**a**) and CCR2^RFP/RFP^ mice (**b**) following acute DSS-induced colitis. **c** Linear regressions between seizure threshold and neutrophil frequencies in the blood of clodronate-treated mice following acute DSS-induced colitis. **d** Latencies to seizure onset (left) and generalization (right) in α-Ly6G-treated mice with acute DSS-induced colitis. **e** MFI of TNFα expressed by monocytes and neutrophils in the blood, and monocytes, neutrophils, and microglia in the brains of control mice and those with acute DSS-induced colitis. **f** Latencies to seizure onset (left) and generalization (right) in α-TNFα-treated mice following acute DSS-induced colitis. *n* = 8 mice/group for flow cytometry analysis, representative of 3–5 replicate experiments. Seizure threshold data represent the combined analysis of 3–4 experiments containing 8–15 mice/condition/experiment. ns=not significant, *p < 0.05, **p < 0.01, ****p < 0.0001

We next investigated the roles of monocytes and neutrophils in colitis-induced neuronal hyperexcitability by measuring seizure threshold following depletion of one or the other of these cell types. Monocyte depletion using clodronate had no impact on seizure threshold following acute DSS-induced colitis (Fig. 3a) despite significant depletion (Supplemental Figure 3a-b). Additionally, there was no change in seizure threshold in colitic CCR2^RFP/RFP^ mice, in which the *Ccr2* locus is replaced with RFP, impairing monocyte migration (Fig. 3b). CCR2^RFP/+^ mice were used as controls. This finding confirms that monocytes do not play a major pathogenic role in colitis-induced neuronal hyperexcitability. By contrast, we observed a strong negative correlation between blood neutrophil frequency and seizure threshold in clodronate-treated mice (Fig. 3c). We then directly evaluated the role of neutrophils by administering α-Ly6G depleting antibodies intraperitoneally from days 6 to 14 following DSS colitis induction. Control mice received equivalent doses of isotype IgG2a antibody on the same dosing schedule. Despite modest neutrophil depletion of 65% (Supplemental Figure 3c-d), we observed a 50% rescue in seizure threshold following neutrophil depletion (Fig. 3d), demonstrating a neuropathogenic role for neutrophils. To determine the mechanism by which neutrophils contribute to colitis-induced neuronal hyperexcitability, we evaluated their production of TNFα, which is known to enhance excitability [57–60]. Based on flow cytometric analysis, neutrophils produced more TNFα than blood and brain monocytes and microglia in colitic mice (Fig. 3e). Furthermore, on a per-cell basis, brain-infiltrating neutrophils produced more TNFα in colitic mice than in healthy mice (Fig. 3e). We assessed the role of TNFα in mediating neuronal hyperexcitability during colitis by administering neutralizing α-TNFα antibody intraperitoneally from days 6 to 14 following DSS colitis induction. Control mice received an equivalent dose of isotype IgG1 antibody on the same dosing schedule. TNFα neutralization was sufficient to improve seizure threshold by 55–60% in colitic mice (Fig. 3f). These findings suggest that the pathogenic role of neutrophils in mediating neuronal hyperexcitability following acute colitis is attributable to their production of TNFα.

### Deconvolution, gene expression, and pathway analysis of resected brain tissue from patients with epilepsy reveal significant overlap between neuronal hyperexcitability and inflammatory pathologies

**Fig. 4:**
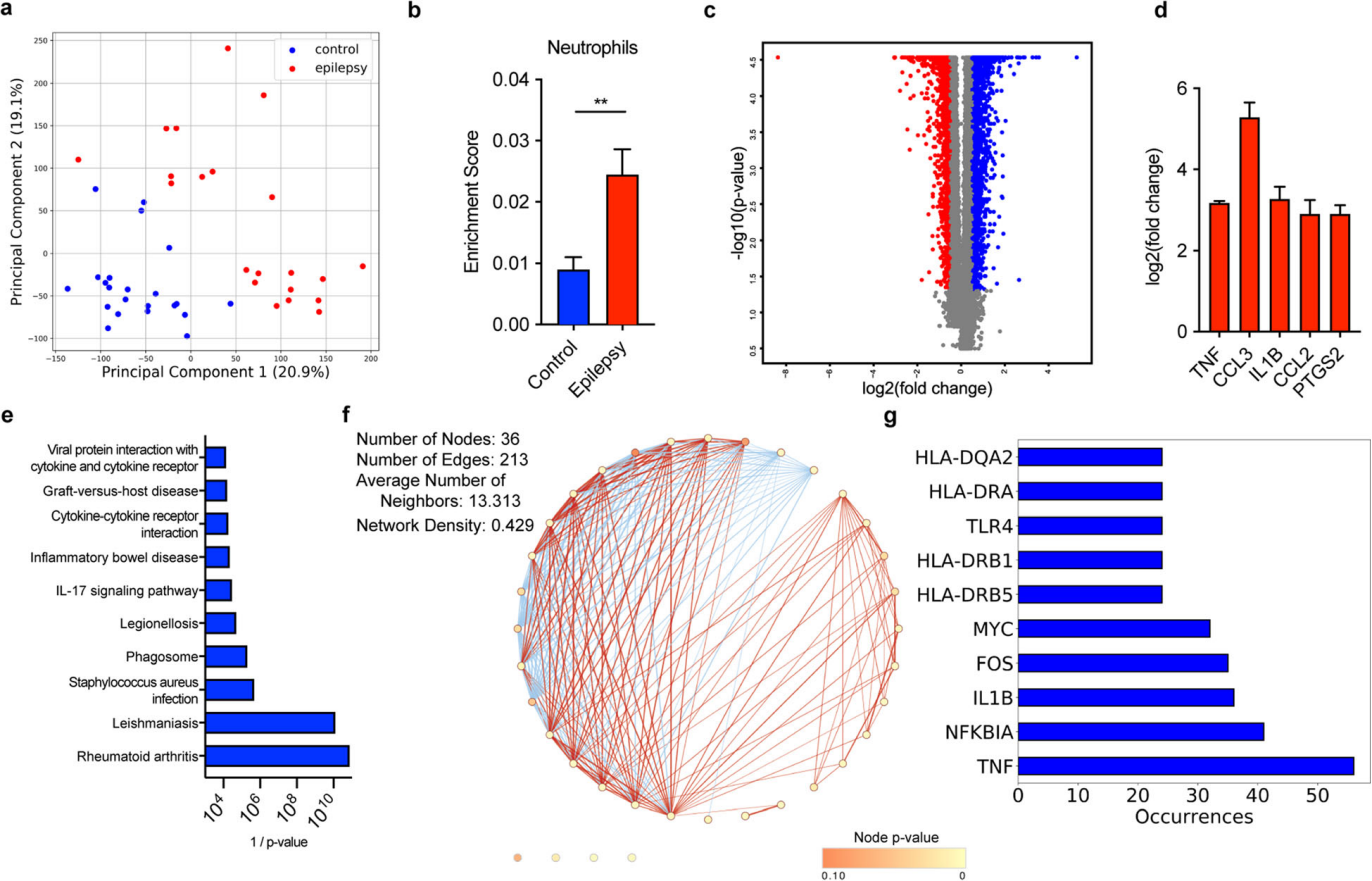
Analysis of human epileptic brain tissue reveals significant overlap between neuronal hyperexcitability and inflammatory pathologies. **a** PCA on gene expression profiles from hippocampal tissue obtained from 21 epileptic patients and 22 healthy controls. **b** CIBERSORTx enrichment score for neutrophils. **c** Volcano plot depicting differentially expressed genes [log2(fold change) > 1, *p*-value < 0.05]. **d** Statistically significant differences in expression of *TNF*, *CCL3*, *IL1B*, *CCL2*, and *PTGS2* between epileptic and control hippocampal tissue. **e** Statistical significance of the differences in expression of the top ten overrepresented KEGG pathways among differentially regulated genes. **f** Network diagram portraying connectivity between KEGG gene sets (nodes), where edges represent shared genes. Red edges denote the presence of *TNF* in the shared gene list between both pathways. **g** Quantification of occurrences where a given gene appears on a shared gene list (edge). Top ten most frequently occurring genes are shown

Having established that neutrophils and TNFα play a critical role in neuronal hyperexcitability in colitic mice, we sought to determine whether this pathophysiology might generalize to humans with neuronal hyperexcitability. Brain specimens from IBD patients are unavailable, and it is also unknown which brain regions are hyperexcitable in IBD patients, so we did not use them for this analysis. Instead, we analyzed a publicly available microarray dataset comparing hyperexcitable brain tissue resected from patients with epilepsy to histologically normal tissue from post-mortem patients with no known neurological disease [61], which enabled us to study tissue that is known to be hyperexcitable. Principal component analysis (PCA) on gene expression profiles revealed a clear separation between epileptic and healthy brain tissue (Fig. 4a). Using CIBERSORTx deconvolution [44], we identified a 2-fold enrichment of neutrophils in epileptic brain tissue compared to healthy brain tissue (Fig. 4b). Differential gene expression analysis (Fig. 4c, Supplemental Table 1) revealed that *TNF*, *CCL3*, *IL1B*, *CCL2*, and *PTGS2* were among the most highly upregulated (>8-fold) genes (Fig. 4d). CCL3 is involved in neutrophil chemotaxis, and MCP-1/CCL2 is involved in monocyte chemotaxis. PTGS2, TNFα, and IL-1β are highly upregulated by inflammatory neutrophils. These findings mirror the neuroinflammatory phenotype observed in colitic mice. Finally, we used overrepresentation analysis to compare the upregulated genes to gene sets from the Kyoto Encyclopedia of Genes and Genomes (KEGG), which revealed that the majority of gene sets overrepresented in epileptic brain tissue were related to those seen in autoinflammatory diseases (including IBD), immune pathways, and infection responses (Fig. 4e, Supplemental Table 2). Gene ontology (GO) analysis confirmed an enrichment in immune pathways (Supplemental Table 3). Network analysis revealed a high level of connectivity between the majority of gene sets (Fig. 4f), suggesting that common pathways underlying many different inflammatory disorders may contribute to neuronal hyperexcitability. *TNF* was the most frequently occurring gene on the edges of the network (Fig. 4f, g, Supplemental Table 4), indicating that it may be a prime target for the prevention or treatment of neuronal hyperexcitability in the context of, and potentially independent of, an underlying peripheral autoinflammatory disease.

## Discussion

Patients with chronic inflammatory disorders frequently experience neurological symptoms related to neuronal hyperexcitability such as psychiatric disorders and epilepsy [1–7]. However, the mechanisms by which peripheral inflammation contributes to the development of these symptoms are poorly understood. Previous studies that have attempted to uncover these mechanisms have largely relied on mouse models of neurological disease, such as spontaneous seizures, neurodegeneration, stroke, and traumatic brain injury [12–18]. These models do not allow for the uncoupling of the impact of neurological pathology on inflammation from the role of peripheral inflammation in neurological dysfunction. Other studies have utilized models of peripheral inflammation that do not closely mimic human autoinflammatory disorders, such as systemic LPS administration [19–24]. Here, we instead utilized three mouse models of colitis, as IBD has been robustly associated with neurological symptoms [2, 4, 8, 30–42]. We mainly relied on the acute DSS model, as its short duration enabled mechanistic studies that would not have been feasible in the chronic models, which develop over 6–8 weeks. Nonetheless, we observed neuronal hyperexcitability in mice with chronic DSS- and TCT-induced colitis, as well as in mice with acute DSS colitis. Furthermore, we detected neuroinflammation in these mice with little to no microglia activation, as well as the presence of brain-infiltrating monocytes and neutrophils. Finally, we discovered a role for neutrophils and TNFα in mediating neuronal hyperexcitability in mice with autoinflammation.

Although epithelial barrier disruption and microbial translocation play key roles in all three models of colitis, since the downstream inflammatory processes are somewhat distinct, colitis may contribute to neuroinflammation and neuronal hyperexcitability via distinct mechanisms in each model. For instance, T cells are not required for the induction of DSS-induced colitis, but they are the primary drivers of TCT-induced colitis [49]. While not required for induction, a T helper 1 (Th1)-polarized immune response has been observed during acute DSS-induced colitis, whereas a mixed Th1/Th2 response is seen during chronic DSS-induced colitis [49]. Conversely, Th1- and Th17-polarized responses are observed during TCT-induced colitis [49]. Despite these differences, our study showed that the peripheral inflammatory responses they generate in the brain and plasma are similar.

While our data point to important roles of neutrophils and TNFα in colitis-induced neuroexcitability, other cells and inflammatory mediators likely also contribute. TNFα may be produced by other leukocytes during colitis, and neutrophils may produce additional factors that contribute to hyperexcitability. For instance, we observed increases in IL-1β and IL-6 in the brains of colitic mice (Fig. 2c), both of which are produced by neutrophils and have been shown to exert neuroinflammatory and neuroexcitatory effects [59, 62–67]. Notably, IL-1β was also one of the most highly upregulated genes in the brains of patients with epilepsy (Fig. 4d). In addition to the production of neuroexcitatory cytokines, neutrophils may contribute indirectly to neuronal hyperexcitability by producing chemokines to recruit other inflammatory cells. Both CCL2 and CCL3, which were highly upregulated in epileptic brain tissue (Fig. 4d), are produced by neutrophils and participate in the recruitment of monocytes and other bone marrow-derived cells. Indeed, the CCL2/CCR2 axis has previously been implicated in inflammation-induced seizure enhancement [17]. Additionally, our studies do not determine whether neutrophils, TNFα, or other mediators are acting exclusively in the brain parenchyma, or whether their effects on vascular tissue and interactions with the enteric nervous system and/or vagus nerve may also be contributing to hyperexcitability. Finally, it is possible that other cells, including microglia, may be involved in spite of the lack of evidence for this in our experiments. Future studies investigating additional mechanisms by which colitis induces neuronal hyperexcitability are necessary for a more complete understanding of the pathological effects of the gut and other forms of peripheral inflammation on neuroinflammation and neuronal dysregulation.

Although it is unlikely that neutrophils and TNFα are the sole mediators of neuronal hyperexcitability in the setting of inflammatory disease, our findings suggest that TNFα blocking agents may be beneficial for the large number of patients with such disorders who experience neurological and psychiatric symptoms. TNFα enhances excitability by increasing glutamate release, regulating AMPA cell surface trafficking to enhance glutamate responses, and causing endocytosis of GABA_A_ receptors [57–60]. It also activates vascular endothelial cells [68, 69] and impairs BBB integrity [70, 71], both of which contribute to neuroinflammation. Furthermore, TNFα inhibition has been shown to improve the sense of well-being, sensory function, and cognitive processing in patients with IBD [72]. Because neuroinflammation occurs in many neurological and psychiatric disorders in the absence of a diagnosed inflammatory condition [25–29], TNFα inhibitors may also prove efficacious in patients without an underlying autoinflammatory disease. Indeed, TNFα has been implicated in major depressive disorder but has not yet been evaluated therapeutically in patients with this disorder [73]. Nonetheless, further studies are essential for understanding the mechanisms by which TNFα contributes to neuronal hyperexcitability in the context of colitis, as TNFα inhibition has in some cases been associated with enhanced excitability and neurodegeneration, especially in the context of demyelinating disorders [59, 63, 74]. Differences between the central and peripheral functions of TNFα, as well as signaling via TNF receptor 1 (TNFR1) versus TNFR2, may be responsible for the contrasting roles of TNFα in different neurological diseases [59, 63, 74].

While our studies reveal a mechanism by which intestinal inflammation results in neuronal hyperexcitability, future studies examining the neuroinflammatory phenotype of mice with other models of autoinflammation such as rheumatoid arthritis or diabetes would be beneficial for understanding whether there is overlap in the mechanisms by which peripheral inflammation contributes to neurological dysfunction, or whether neurological symptoms in different autoimmune disorders are the result of processes specific to each disease. Our gene expression and pathway analysis of human epileptic brain tissue suggests that hyperexcitability of diverse etiology may result from similar inflammatory mechanisms, as the transcriptional signature of hyperexcitable brain tissue had significant overlap with gene sets associated with autoinflammatory disorders. Furthermore, there was a high level of connectivity between the vast majority of these gene sets, indicating that pathways common to a large number of autoinflammatory disorders may contribute to neuronal hyperexcitability, rather than each disorder causing neurological dysfunction via a unique mechanism. TNFα was the highest occurring gene on the edges of the network, highlighting its important role in immune pathologies associated with neurological dysfunction.

## Conclusions

Our results reveal neutrophils and TNFα as central regulators of neuronal hyperexcitability of diverse etiology. Since existing treatments for neurological disorders largely target neurons and not inflammation, these treatments therefore fail to address this component. Furthermore, these drugs have serious side effect profiles, and many patients are unresponsive to them. Because treatments targeting inflammatory mediators do not directly target neuronal function but may address a major component of the underlying pathology, such treatments would be expected to have superior efficacy as well as side effect profiles. Thus, there is a strong rationale for evaluating anti-inflammatory agents, including TNFα inhibitors, for the treatment of neurological and psychiatric symptoms present in, and potentially independent of, a diagnosed inflammatory disorder.

## Supplementary Information

**Additional file 1: Supplemental Figure 1.** Linear regressions between cytokine transcription and *Tjp1* and *Cldn5*. *n* = 5-10 mice/group, representative of 3-5 replicate experiments. **Supplemental Figure 2.** Frequencies of immune cell populations in the brains of mice following acute DSS-induced colitis. Flow cytometric quantification of **a**) macrophages (CD45^+^CD11b^+^Ly6G^-^Ly6C^-^MHCII^+^), **b**) dendritic cells (CD45^+^CD11b^+^Ly6G^-^CD11c^+^), **c**) B cells (CD45^+^CD11b^-^CD3^-^CD19^+^), and **d**) T cells (CD45^+^CD11b^-^CD3^+^). *n* = 5-10 mice/group, representative of 3-5 replicate experiments. ns=not significant, *p < 0.05. **Supplemental Figure 3.** Depletion efficiency of monocytes and neutrophils in mice following acute DSS-induced colitis. **a-b**) 200mL of clodronate-loaded liposomes was administered on days 6-14 during acute DSS-induced colitis. **a**) Flow cytometric quantification of monocytes (CD45^+^CD11b^+^Ly6G^-^Ly6C^+^MHCII^-^) in colitic mouse blood following clodronate treatment. **b**) Percent of monocytes in colitic mouse blood following clodronate treatment normalized to the mean percent of monocytes in colitic mice treated with PBS-loaded liposomes. **c-d**) 500mg α-Ly6G antibody was administered on days 6-14 during acute DSS-induced colitis induction. **c**) Flow cytometric quantification of neutrophils (CD45^+^CD11b^+^Ly6G^+^) in colitic mouse blood following α-Ly6G antibody treatment. **d**) Percent of neutrophils in colitic mouse blood following α-Ly6G antibody treatment normalized to the mean percent of neutrophils in colitic mice treated with isotype IgG2a antibody. Data are representative of 3-4 combined experiments with 5-15 mice/group/experiment. *p < 0.05, ****p < 0.0001. **Supplemental Figure 4**. Representative images of *in vivo* α-CD45 staining of border-associated immune cells. **a**) Immunofluorescence images of the choroid plexus in healthy and colitic mice following *in vivo* staining of border regions with an α-CD45 antibody and subsequent *ex vivo* α-CD45 staining of all leukocytes. Parenchymal cells (i.e. microglia) do not stain positive for the *in vivo*-administered α-CD45, but border-associated cells (i.e. those in the choroid plexus) stain for both the *in vivo*- and *ex vivo*-administered α-CD45. **b**) Representative flow cytometry plots of *in vivo*- and *ex vivo*-administered α-CD45 staining of microglia (CD45^lo^CX3CR1^hi^CD11b^+^) and neutrophils (CD45^+^CD11b^+^Ly6G^+^) in the brains of healthy and colitic mice. Microglia (parenchymal cells) are not stained by the *in vivo*-administered α-CD45 in either condition. 031821 J Neuroinflammation Tables.pdf. **Supplementary Tables.**

## Data Availability

All data generated and analyzed during this study are included in this published article and its supplementary information. The public microarray dataset from resected brain tissue from human epilepsy patients is available in the ArrayExpress repository: https://www.ebi.ac.uk/arrayexpress/experiments/E-MTAB-3123/

## Abbreviations

DSS: Dextran sodium sulfate
GABA: γ-Aminobutyric acid
GO: Gene ontology
IBD: Inflammatory bowel disease
LPS: Lipopolysaccharide
PCA: Principal component analysis
TCT: T cell transfer
