# Supplementary material for: Brain profiling in murine colitis and human epilepsy reveals neutrophils and TNFα as mediators of neuronal hyperexcitability
Source: J Neuroinflammation. 2021 Sep 12;18:199. doi: 10.1186/s12974-021-02262-4 (PMC8436533; doi:10.1186/s12974-021-02262-4)
Supplement: Supplementary file 1 — Additional file 1: Supplemental Figure 1. Linear regressions between cytokine transcription and Tjp1 and Cldn5. n = 5-10 mice/group, representative of 3-5 replicate experiments. Supplemental Figure 2. Frequencies of immune cell populations in the brains of mice following acute DSS-induced colitis. Flow cytometric quantification of a) macrophages (CD45+CD11b+Ly6G-Ly6C-MHCII+), b) dendritic cells (CD45+CD11b+Ly6G-CD11c+), c) B cells (CD45+CD11b-CD3-CD19+), and d) T cells (CD45+CD11b-CD3+). n = 5-10 mice/group, representative of 3-5 replicate experiments. ns=not significant, *p < 0.05. Supplemental Figure 3. Depletion efficiency of monocytes and neutrophils in mice following acute DSS-induced colitis. a-b) 200mL of clodronate-loaded liposomes was administered on days 6-14 during acute DSS-induced colitis. a) Flow cytometric quantification of monocytes (CD45+CD11b+Ly6G-Ly6C+MHCII-) in colitic mouse blood following clodronate treatment. b) Percent of monocytes in colitic mouse blood following clodronate treatment normalized to the mean percent of monocytes in colitic mice treated with PBS-loaded liposomes. c-d) 500mg α-Ly6G antibody was administered on days 6-14 during acute DSS-induced colitis induction. c) Flow cytometric quantification of neutrophils (CD45+CD11b+Ly6G+) in colitic mouse blood following α-Ly6G antibody treatment. d) Percent of neutrophils in colitic mouse blood following α-Ly6G antibody treatment normalized to the mean percent of neutrophils in colitic mice treated with isotype IgG2a antibody. Data are representative of 3-4 combined experiments with 5-15 mice/group/experiment. *p < 0.05, ****p < 0.0001. Supplemental Figure 4. Representative images of in vivo α-CD45 staining of border-associated immune cells. a) Immunofluorescence images of the choroid plexus in healthy and colitic mice following in vivo staining of border regions with an α-CD45 antibody and subsequent ex vivo α-CD45 staining of all leukocytes. Parenchymal cells (i.e. microglia) do not s [file 12974_2021_2262_MOESM1_ESM.pdf]

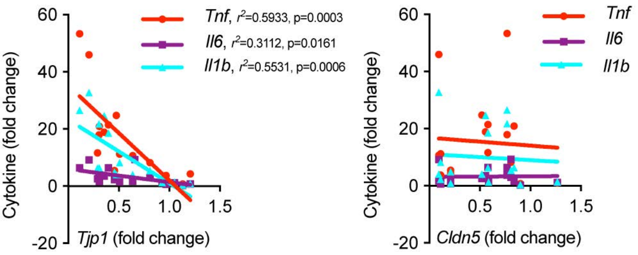

Tnf, r2=0.5933, p=0.0003
Il6, r2=0.3112, p=0.0161
Il1b, r2=0.5531, p=0.0006
Cytokine (fold change)
Tjp1 (fold change)
60
40
20
0
-20
0.5
1.0
1.5
Tnf
Il6
Il1b
Cytokine (fold change)
Cldn5 (fold change)

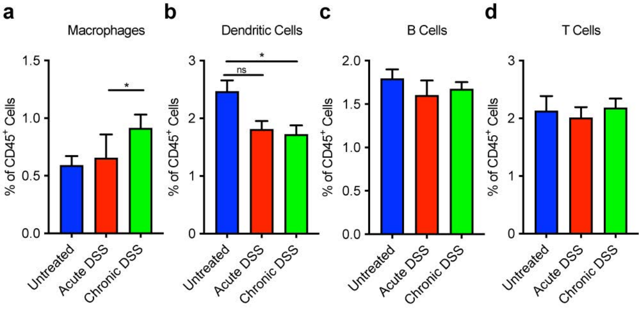
a
Macrophages
% of CD45⁺ Cells
1.5
1.0
0.5
0.0
*
Untreated
Acute DSS
Chronic DSS
b
Dendritic Cells
% of CD45⁺ Cells
3
2
1
0
ns
*
Untreated
Acute DSS
Chronic DSS
c
B Cells
% of CD45⁺ Cells
2.0
1.5
1.0
0.5
0.0
Untreated
Acute DSS
Chronic DSS
d
T Cells
% of CD45⁺ Cells
3
2
1
0
Untreated
Acute DSS
Chronic DSS

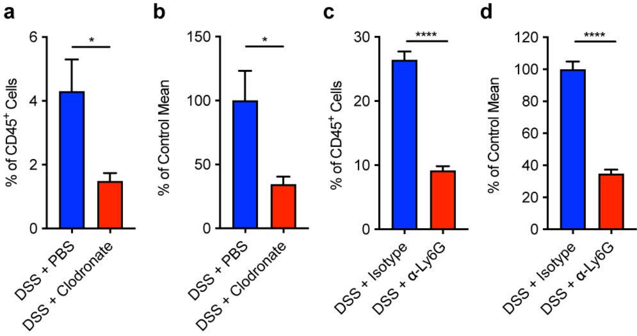

a
b
c
d
*
*
****
****
% of CD45+ Cells
% of Control Mean
% of CD45+ Cells
% of Control Mean
0
2
4
6
0
50
100
150
0
10
20
30
0
20
40
60
80
100
120
DSS + PBS
DSS + Clodronate
DSS + PBS
DSS + Clodronate
DSS + Isotype
DSS + α-Ly6G
DSS + Isotype
DSS + α-Ly6G

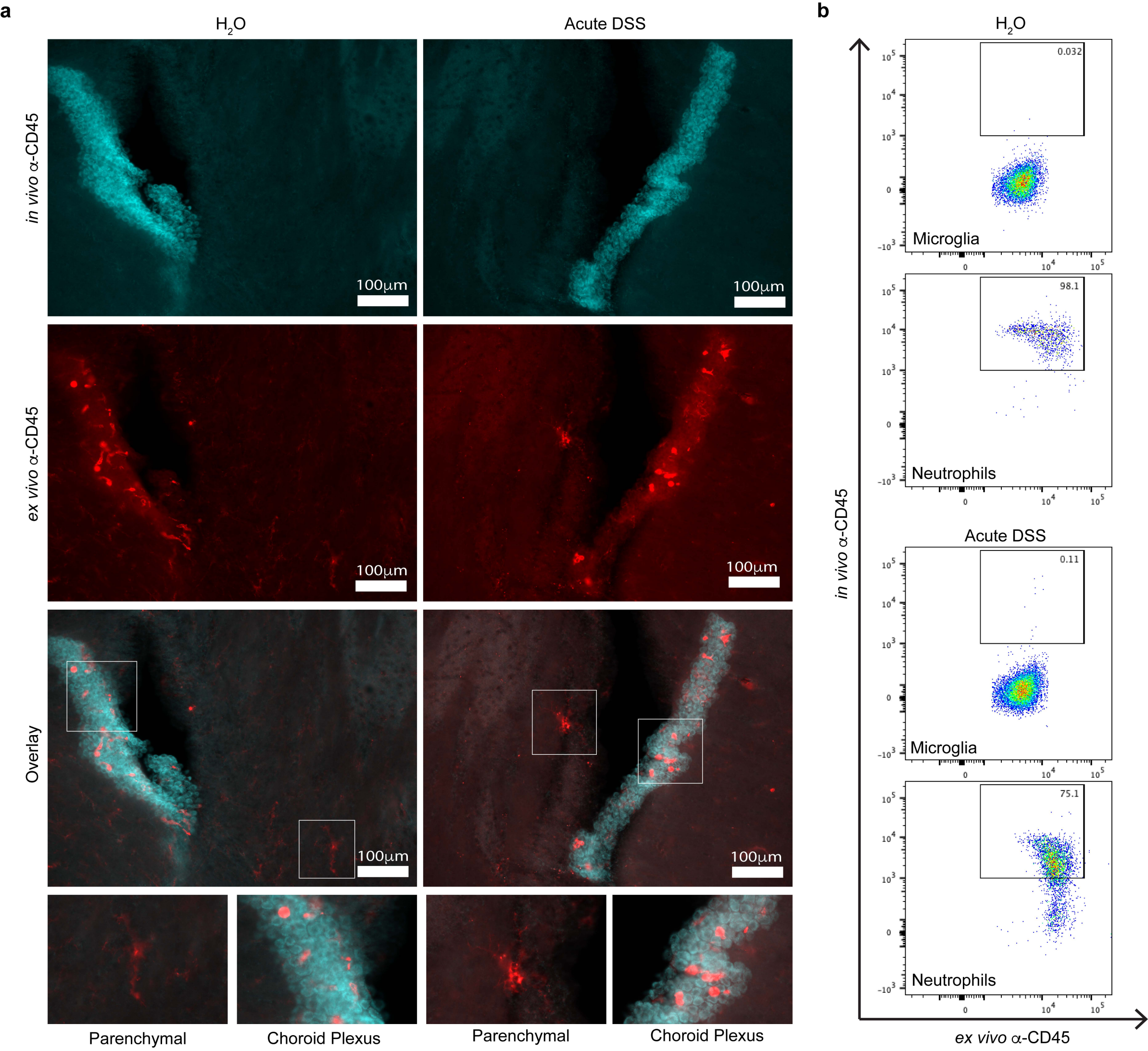
a
H2O
Acute DSS
in vivo α-CD45
ex vivo α-CD45
Overlay
100μm
Parenchymal
Choroid Plexus
Parenchymal
Choroid Plexus
b
H2O
Microglia
0.032
Neutrophils
98.1
Acute DSS
Microglia
0.11
Neutrophils
75.1
in vivo α-CD45
ex vivo α-CD45

Supplemental Table 1. Complete list of genes that are significantly down- or up-regulated in hippocampal tissue from epileptic patients compared to controls.

Downregulated Genes	p_value	log2(fold change)	Upregulated Genes	p_value	log2(fold change)
TTR	2.93E-05	-8.380821784	CCL3	2.93E-05	5.285439211
SCGN	2.93E-05	-3.058893689	CH25H	2.93E-05	3.55741009
CLIC6	2.97E-05	-3.046921047	TNF	2.93E-05	3.430419097
FNDC1	2.93E-05	-3.023269779	IL1B	2.93E-05	3.264986734
C11orf88	8.43E-05	-2.795859283	CCL2	2.93E-05	2.903423966
CABP7	2.93E-05	-2.708396442	PTGS2	2.93E-05	2.897240426
C9orf117	4.73E-05	-2.689659879	DHRS9	2.93E-05	2.847596102
COL25A1	2.93E-05	-2.662003536	FOSB	2.95E-05	2.697106574
DCDC1	2.94E-05	-2.590744853	XIST	0.03537679	2.647084213
CABP7	2.93E-05	-2.564904848	CCL8	3.54E-05	2.628307006
KLK1	6.75E-05	-2.490050854	TMEM233	2.93E-05	2.612352499
DCDC1	2.94E-05	-2.465938398	MIR17HG	2.93E-05	2.557042415
NTS	0.000290431	-2.457989644	FREM3	4.98E-05	2.416839742
FNDC1	3.11E-05	-2.388355457	GLI1	2.93E-05	2.308011315
MCOLN3	2.93E-05	-2.380821784	PTGS2	2.93E-05	2.303050085
C11orf88	0.0005547	-2.351074441	TMEM215	3.84E-05	2.242754922
MAS1	2.93E-05	-2.351074441	NAT16	2.93E-05	2.180466161
C6orf118	2.93E-05	-2.336427665	SEMA3A	2.98E-05	2.116697443
FAM81B	0.000107459	-2.321928095	IL1RN	2.93E-05	2.034215715
FAM183A	5.25E-05	-2.314732593	PPBP	0.0002205	2.032453537
TSPAN18	2.95E-05	-2.307572802	SOCS3	3.22E-05	1.947853143
STOML3	3.27E-05	-2.293358943	MCHR2	0.00806785	1.931683057
SHISA6	2.94E-05	-2.265344567	CSF2RA	2.93E-05	1.918004519
LINC00314	3.15E-05	-2.258425153	C1QL4	2.94E-05	1.910348915
WNK4	2.95E-05	-2.224317298	SMYD1	0.001305	1.904965719
DNAH11	2.94E-05	-2.210896782	MCHR2	0.00066639	1.900335509
CHRNA1	2.93E-05	-2.210896782	SEMA3A	2.93E-05	1.898788797
SHISA6	2.94E-05	-2.204233052	WISP1	2.93E-05	1.892973815
ADRA1D	2.93E-05	-2.204233052	SMPX	0.00258729	1.891808001
WIPF3	2.93E-05	-2.204233052	ANKRD22	0.00031849	1.871055075
PRLR	3.39E-05	-2.177881725	FBXO40	0.00248285	1.860764203
C9orf171	9.88E-05	-2.171368418	EGR2	2.93E-05	1.847596102
CDRT15L2	3.01E-05	-2.029146346	ZFP36	8.43E-05	1.845188935
CACNG8	2.93E-05	-2.011587974	IPCEF1	5.25E-05	1.834711216
DNAI2	0.000349687	-1.977099598	TMEM159	2.95E-05	1.797843067
SPAG17	3.15E-05	-1.977099598	FOXP2	3.39E-05	1.765959006
EFCC1	2.93E-05	-1.977099598	SEMA3D	2.93E-05	1.763836459
C4orf22	2.93E-05	-1.965784285	MKX	3.13E-05	1.746742716
POSTN	4.51E-05	-1.960159735	IRAK2	2.93E-05	1.745452483
KCNE1	2.93E-05	-1.948975997	CX3CR1	2.93E-05	1.744161096
SHISA6	2.93E-05	-1.943416472	B3GNT5	2.93E-05	1.742437445
ADGB	3.19E-05	-1.899695094	TNFRSF11A	4.05E-05	1.741574847
BCL11B	2.93E-05	-1.883635243	KLHL1	0.00474318	1.740711733
CHRNA1	3.08E-05	-1.862496476	THBS1	4.31E-05	1.737686761
SPHKAP	2.93E-05	-1.857259828	DPP4	4.73E-05	1.691980751
COL5A2	3.03E-05	-1.852042119	GPR6	0.01244972	1.689746438
BHLHE22	2.94E-05	-1.846843212	AC004383.4	2.93E-05	1.685267407
RP11-2E17.1	2.93E-05	-1.831357964	FOS	0.0002205	1.683471893
PRKCG	2.93E-05	-1.826232932	C3	2.95E-05	1.644779219
NTF3	2.93E-05	-1.821126042	ACY3	2.93E-05	1.63784206
RGS14	2.93E-05	-1.805912948	SLC17A6	0.03987572	1.63691458
SLC47A2	0.035376791	-1.800877358	HLA-DQA2	8.43E-05	1.631337144
SAG	9.12E-05	-1.800877358	TAC3	3.06E-05	1.629473197
TNNT2	3.62E-05	-1.790858602	TAL1	2.93E-05	1.621524444
COL5A2	2.97E-05	-1.780908942	SOD2	0.00020109	1.613531653
COL2A1	2.99E-05	-1.775959726	BTG2	2.93E-05	1.60928218
COL25A1	2.99E-05	-1.775959726	FPR1	3.03E-05	1.579662897
NEUROD6	2.93E-05	-1.756330919	BCL2A1	0.00060839	1.573859222
FBP2	3.84E-05	-1.751465164	GPR34	2.93E-05	1.573374526
HPCA	2.93E-05	-1.746615764	OLR1	3.22E-05	1.572889668
SMPD3	2.93E-05	-1.74178261	KLF6	2.95E-05	1.570462931
SYNGAP1	2.93E-05	-1.736965594	FCAR	0.00016685	1.562669826
SLC47A1	3.08E-05	-1.727379545	MMRN1	3.08E-05	1.554834396
CLMP	2.93E-05	-1.727379545	RP11-191L9.4	0.00087605	1.537544572
LINC00314	3.22E-05	-1.722610301	CYP4F2	2.94E-05	1.5360529
SLC24A3	2.98E-05	-1.713118852	PAX2	0.0012496	1.5290713

CPLX2	2.93E-05	-1.713118852	SLN	0.00598002	1.525066592
CCDC114	0.000349687	-1.694321257	SPTLC3	2.93E-05	1.522055749
SLC24A3	3.11E-05	-1.694321257	RTP1	2.93E-05	1.519541905
Downregulated	Upregulated				
PPM1E	2.93E-05	-1.685013515	IKZF1	3.11E-05	1.516519498
POSTN	5.25E-05	-1.675765438	FMN1	3.39E-05	1.508935662
MCOLN3	2.93E-05	-1.675765438	APOC4	0.00046195	1.507414099
CXXC11	3.01E-05	-1.666576266	HLA-DRA	0.00024196	1.504874589
SPAG6	0.000107459	-1.662003536	CD53	2.93E-05	1.502839758
SKA3	3.22E-05	-1.662003536	HPGDS	2.93E-05	1.496717988
C9orf135	3.01E-05	-1.652901329	IER3	3.08E-05	1.496206666
SPHKAP	2.94E-05	-1.639354798	RXFP1	0.00405021	1.491596594
DNAI2	3.62E-05	-1.634867407	NMU	0.02435809	1.485426827
ITGA8	2.93E-05	-1.63039393	ARHGAP36	0.00079975	1.475603745
TEKT1	3.84E-05	-1.621488377	IPCEF1	9.12E-05	1.463099267
CERKL	2.93E-05	-1.60823228	CBLN4	0.00079975	1.462575888
SPHKAP	2.94E-05	-1.603840511	SYTL2	4.73E-05	1.46048047
KIAA1257	2.93E-05	-1.603840511	PYDC2	0.00136518	1.454175893
CDHR4	0.000461948	-1.59946207	SYTL2	4.14E-05	1.45206823
TOMM34	2.93E-05	-1.59946207	CD84	2.98E-05	1.449957484
CLSPN	2.96E-05	-1.595096878	RNU4ATAC	2.93E-05	1.449957484
NRP1	2.96E-05	-1.582079992	CSF1R	2.94E-05	1.449429314
RP11-408E5.5	9.12E-05	-1.573466862	RXFP1	0.00749242	1.446785562
ALDH1A2	2.93E-05	-1.573466862	TINCR	6.75E-05	1.444136957
CLMP	2.93E-05	-1.569179503	LAPTM5	2.96E-05	1.439357178
CCNJL	2.93E-05	-1.552156356	RGS18	2.98E-05	1.430820497
DCC	2.93E-05	-1.543719518	F2R	2.93E-05	1.42921423
MYLK3	0.000290431	-1.535331733	VSIG4	3.98E-05	1.42867841
CCDC108	7.80E-05	-1.535331733	CLEC7A	3.11E-05	1.427606173
STC2	3.62E-05	-1.535331733	TLR7	2.97E-05	1.427606173
CLDN2	0.000166853	-1.531156057	MPEG1	2.93E-05	1.426533138
COL25A1	0.000107459	-1.522840789	HTR1D	0.00072996	1.419538892
SMPD3	2.93E-05	-1.522840789	PNOC	0.00104668	1.41467678
C1QTNF9	0.000290431	-1.514573173	TNIP3	8.43E-05	1.413594082
CARTPT	0.000220502	-1.514573173	MYC	2.93E-05	1.412510571
DNAH12	4.98E-05	-1.506352666	DONSON	2.93E-05	1.407080775
NRP1	2.94E-05	-1.498178735	SUCNR1	0.00015222	1.404903122
CNIH2	2.93E-05	-1.498178735	RP11-747H7.3	0.00024196	1.371280054
FOXJ1	0.004384695	-1.49410907	MIR222	2.95E-05	1.366811773
CAMK2A	2.93E-05	-1.49410907	IER2	3.06E-05	1.36232961
ALDH1A2	2.95E-05	-1.490050854	CD83	2.93E-05	1.360083296
SAMD3	2.95E-05	-1.486004021	NFKBID	3.54E-05	1.355580147
FAM43A	2.93E-05	-1.481968507	ST6GAL1	2.93E-05	1.352758525
AFF2	2.93E-05	-1.477944251	RP11-747H7.3	6.75E-05	1.352193538
FEZF2	2.93E-05	-1.473931188	PRDM1	2.93E-05	1.34879896
KIT	2.93E-05	-1.469929258	P2RY12	2.93E-05	1.347098671
NLGN4Y	0.027655557	-1.465938398	AQP9	0.00136518	1.346531462
SIPA1L3	2.93E-05	-1.461958547	RP11-747H7.3	0.0004217	1.345396375
LDLRAD2	6.32E-05	-1.457989644	MEF2C	3.84E-05	1.343123519
ONECUT2	2.94E-05	-1.457989644	C1QB	3.27E-05	1.341416524
VWA5B1	0.000265126	-1.454031631	SCARA5	0.00066639	1.336283388
DOC2B	2.93E-05	-1.454031631	SYK	2.93E-05	1.333423734
SYNE1	2.93E-05	-1.446148032	AIF1	3.13E-05	1.332278283
RP11-122F24.1	0.000303828	-1.442222329	ATF3	7.80E-05	1.331705216
CAMK2A	2.93E-05	-1.434402824	EGR1	4.98E-05	1.331131922
KCNIP2	2.93E-05	-1.430508908	RP11-150O12.1	4.73E-05	1.328262029
SPATA18	2.97E-05	-1.426625474	C1QA	3.11E-05	1.328262029
CHST8	2.93E-05	-1.426625474	WISP1	3.54E-05	1.326537348
PRSS12	2.93E-05	-1.422752464	AC144521.1	0.00405021	1.323658291
TOMM34	2.93E-05	-1.422752464	ALOX5AP	2.94E-05	1.319617934
VEGFA	2.93E-05	-1.422752464	CYP4F3	0.00598002	1.317882883
WIPF3	2.93E-05	-1.415037499	RGR	0.00029043	1.310921735
KCNJ13	0.000201094	-1.411195433	C19orf77	0.00010746	1.30626224
C9orf135	6.32E-05	-1.411195433	SATB2-AS1	0.00749242	1.305678743
NEUROG2	3.84E-05	-1.411195433	ECHDC3	3.15E-05	1.303926836
SYNGAP1	2.93E-05	-1.411195433	IRF8	2.96E-05	1.300416628
ARMC3	0.00072996	-1.407363571	NUDT10	9.12E-05	1.299244658
IKZF3	2.95E-05	-1.407363571	GAPT	9.12E-05	1.295135249
NRIP3	2.93E-05	-1.40354186	TCAM1P	6.75E-05	1.294547234
PRLR	3.15E-05	-1.399730246	STEAP1	0.00018314	1.293958979

SAMD3	2.94E-05	-1.395928676	TMEM196	0.00060839	1.292781749
CHST8	2.94E-05	-1.395928676	OTOGL	0.00050599	1.292781749
EPHA7	2.96E-05	-1.388355457	FOXP2	0.00024196	1.289244285
WNK4	2.93E-05	-1.388355457	LINC00338	2.93E-05	1.288653864
BHLHE22	2.93E-05	-1.384583703	GPR150	0.00210005	1.287472295
CERKL	2.94E-05	-1.380821784	DPP10	3.57E-05	1.285698126
C17orf72	2.93E-05	-1.373327247	FYB	2.95E-05	1.284514133
PLXNA4	2.93E-05	-1.373327247	GADD45B	0.00013922	1.275603079
ARHGEF25	2.93E-05	-1.373327247	NR4A1	0.00269853	1.272620455
WDR63	3.98E-05	-1.365871442	TLR7	3.98E-05	1.272620455
VEPH1	2.93E-05	-1.365871442	GNB4	2.93E-05	1.271425676
CHGB	2.93E-05	-1.365871442	CFB	2.93E-05	1.270229907
CTB-52I2.8	2.93E-05	-1.365871442	TNFRSF10D	3.33E-05	1.269033146
SEMA6B	2.93E-05	-1.365871442	DOCK8	3.27E-05	1.268434394
HRK	2.93E-05	-1.358453971	TFEC	4.73E-05	1.266636643
APOA2	6.75E-05	-1.354759487	SPINT1	4.51E-05	1.266636643
TESC	2.93E-05	-1.343732465	ADORA3	4.31E-05	1.266636643
BCL11B	2.93E-05	-1.336427665	SETD5	2.93E-05	1.264236151
HTR1A	2.94E-05	-1.329159664	PTCHD1	2.93E-05	1.263034406
NPTXR	2.95E-05	-1.325539348	OSM	3.13E-05	1.261229909
CPLX2	2.93E-05	-1.321928095	PLCH1	2.93E-05	1.259423152
DNAH8	2.93E-05	-1.318325858	AC005062.2	0.00029043	1.258820397
GABRQ	0.008686065	-1.311148256	AC005062.2	0.00031849	1.25761413
SMTNL2	4.51E-05	-1.311148256	3/3/20	0.00050599	1.251567621
NRIP3	3.08E-05	-1.311148256	C3	3.22E-05	1.24610401
MTND2P32	2.93E-05	-1.311148256	FOXP2	6.32E-05	1.243669081
PPP1R9B	2.93E-05	-1.311148256	TNFSF18	9.88E-05	1.243059706
SHANK3	2.93E-05	-1.311148256	TMEM255A	2.97E-05	1.242450074
PLXNA4	2.93E-05	-1.307572802	CXCL1	0.01160103	1.241230036
TSPAN16	3.08E-05	-1.304006187	FOXP2	5.57E-05	1.240008965
TG	3.13E-05	-1.300448367	C6orf141	0.00034969	1.238175419
IRX3	0.013350486	-1.2968993	RP11-65F13.2	2.94E-05	1.236951759
COL6A3	0.00228358	-1.2968993	SECTM1	3.11E-05	1.234501321
CLPSL1	0.002482846	-1.293358943	SAMSN1	2.99E-05	1.23388806
TNNT2	9.88E-05	-1.293358943	RORB	0.0002205	1.230203013
SLC47A1	3.98E-05	-1.289827252	AC005062.2	0.00072996	1.22897257
ARMC3	0.003447984	-1.282789701	OSTN	0.0005547	1.228356954
NSMF	2.93E-05	-1.282789701	LINC00338	2.93E-05	1.227741076
CCDC67	3.72E-05	-1.275786313	HTR2A	0.00868607	1.225891862
RP11-85M11.2	2.93E-05	-1.272297327	C5orf63	2.93E-05	1.225891862
PSD	2.93E-05	-1.272297327	PTCHD1	2.93E-05	1.224657734
PTTG1	3.54E-05	-1.268816758	SIGLEC10	2.95E-05	1.222186307
RP11-122F24.1	0.000505987	-1.261880711	SGK1	0.00013922	1.220949004
METTL7B	0.000116923	-1.261880711	SDPR	9.88E-05	1.220329955
C9orf16	2.93E-05	-1.261880711	SPATA20	0.0037383	1.218471211
PTK2B	2.93E-05	-1.261880711	NUDT4	2.97E-05	1.218471211
SPEF1	2.95E-05	-1.258425153	NCF2	3.72E-05	1.217851097
NSMF	2.93E-05	-1.258425153	TINCR	4.14E-05	1.217230716
PLXNA4	2.93E-05	-1.258425153	TMEM159	3.06E-05	1.215367972
C1orf95	2.93E-05	-1.254977851	RP11-728F11.1	2.93E-05	1.21350282
GRP	0.000241964	-1.251538767	NPY	2.96E-05	1.212880566
DNAI2	3.54E-05	-1.251538767	TC2N	0.00060839	1.212258044
RSPO4	4.73E-05	-1.248107862	NFKBIZ	2.93E-05	1.211635253
IBSP	3.39E-05	-1.248107862	CSGALNACT1	2.93E-05	1.210388864
NRIP3	3.13E-05	-1.248107862	CD84	3.13E-05	1.206643224
CACNG8	2.93E-05	-1.248107862	ADAM28	3.72E-05	1.206018004
RBFOX1	2.93E-05	-1.248107862	HAVCR2	3.62E-05	1.205392513
FAM189A1	2.93E-05	-1.248107862	ABCA6	3.84E-05	1.204140717
NRP1	2.98E-05	-1.244685096	SV2C	0.00029043	1.202887833
LZTS1	2.93E-05	-1.244685096	IER3	3.11E-05	1.202887833
ZFPM2	2.93E-05	-1.241270432	IKZF1	2.93E-05	1.199750857
FAM92B	0.001772074	-1.23786383	NCKAP1L	2.97E-05	1.199122642
KIT	2.97E-05	-1.234465254	IGDCC4	4.98E-05	1.197236355
ROPN1L	3.03E-05	-1.231074664	STMN1	2.94E-05	1.197236355
KIAA1456	2.93E-05	-1.227692025	STRIP2	4.51E-05	1.195977459
SIPA1L3	2.94E-05	-1.224317298	CASP1	2.93E-05	1.194717463
NRP2	2.97E-05	-1.220950447	CBX3P2	2.96E-05	1.193456366
KCNA1	2.93E-05	-1.220950447	RFX4	0.00011692	1.19093086
RSPO3	2.93E-05	-1.217591435	CYBB	3.11E-05	1.19093086
PRND	0.000384174	-1.214240226	M1AP	2.94E-05	1.189666447

SLC47A1	4.14E-05	-1.214240226	SEC24D	3.01E-05	1.188400925
SBK1	2.93E-05	-1.214240226	TAGAP	3.03E-05	1.185866545
BCAS4	2.93E-05	-1.214240226	PLA2G7	4.73E-05	1.184597684
PLA2G4D	3.15E-05	-1.210896782	TTN	3.13E-05	1.184597684
SLC26A10	2.93E-05	-1.210896782	IGSF6	2.99E-05	1.183962835
NEFH	2.95E-05	-1.204233052	THEMIS	0.0012496	1.183327706
WIPF3	2.93E-05	-1.200912694	CTD-2521M24.5	0.00050599	1.183327706
GPR64	0.000876053	-1.19759996	EFNA5	0.00020109	1.183327706
CACNA1I	2.93E-05	-1.19759996	NUDT10	5.57E-05	1.18142064
TTC39C	2.93E-05	-1.19759996	IRAK3	3.13E-05	1.18142064
MTUS2	2.93E-05	-1.19759996	HSPB3	0.00079975	1.178873958
KCNAB2	2.93E-05	-1.19759996	THBS1	9.88E-05	1.176960992
VGLL3	0.001143313	-1.194294815	FAP	2.94E-05	1.176322773
DLGAP3	2.93E-05	-1.194294815	AC011242.6	2.93E-05	1.171847314
ONECUT2	0.000666388	-1.190997225	RP11-792D21.2	0.00034969	1.171206827
PTPRN2	2.93E-05	-1.190997225	PDZRN3	3.98E-05	1.171206827
C1orf173	2.93E-05	-1.190997225	EMP1	0.00292929	1.168642036
MDK	2.93E-05	-1.190997225	RGS10	3.22E-05	1.166715445
NEUROD1	3.46E-05	-1.181149439	EPB41L4B	0.01244972	1.166072676
GRIA1	2.94E-05	-1.181149439	NR4A3	0.00079975	1.164786278
KCNAB1	2.93E-05	-1.177881725	ASXL3	3.01E-05	1.164142649
SPAG8	0.000505987	-1.174621396	TMEM156	0.00013922	1.162854528
ADCY9	2.93E-05	-1.168122759	SNTB2	2.96E-05	1.161565256
SUSD4	3.04E-05	-1.164884385	ADORA3	3.27E-05	1.159629186
SYNE1	2.93E-05	-1.164884385	ALOX5AP	3.33E-05	1.157690514
STXBP5L	2.93E-05	-1.164884385	ADAD2	0.00269853	1.155101558
TOMM34	2.93E-05	-1.161653263	CLDN10	2.94E-05	1.155101558
COL24A1	7.80E-05	-1.158429363	PLCH1	2.93E-05	1.153805336
NEURL1B	2.94E-05	-1.158429363	PLEKHF2	2.93E-05	1.153156788
ALPK1	3.62E-05	-1.152003093	PAH	3.27E-05	1.152507948
RIN1	2.96E-05	-1.152003093	KBTBD8	2.93E-05	1.152507948
EPB41L4A	2.95E-05	-1.152003093	HLA-DRB5	0.0004217	1.151858817
ARHGEF25	2.93E-05	-1.152003093	HLA-DRB1	5.25E-05	1.151209393
GRIN1	2.93E-05	-1.152003093	MSR1	4.98E-05	1.151209393
PKP2	3.01E-05	-1.148800661	CYS1	2.96E-05	1.149909668
CACNA1H	2.96E-05	-1.148800661	APBB1IP	4.51E-05	1.147957881
TNFRSF25	2.93E-05	-1.148800661	GADD45G	0.00149036	1.146655222
HCN2	2.93E-05	-1.148800661	PHYHIPL	2.93E-05	1.145351386
PTTG2	2.93E-05	-1.142417045	GRIK3	7.24E-05	1.144699025
SLC25A23	2.93E-05	-1.142417045	SLC7A7	3.13E-05	1.143393419
CCDC64	2.93E-05	-1.142417045	IL6ST	2.93E-05	1.143393419
CELF3	2.93E-05	-1.142417045	AP3B1	2.93E-05	1.142740172
ARHGAP44	2.93E-05	-1.142417045	MCL1	2.97E-05	1.14208663
WASL	2.93E-05	-1.139235797	CLEC9A	0.00020109	1.139469495
DNAAF1	2.97E-05	-1.13606155	EIF4E	0.02435809	1.13553487
NDUFA13	2.93E-05	-1.13606155	SASH3	2.96E-05	1.134878054
EHF	2.96E-05	-1.13289427	CBLN4	0.0037383	1.133563526
ATP2B3	2.93E-05	-1.13289427	LILRB4	0.00066639	1.133563526
NCS1	2.93E-05	-1.13289427	TREM2	0.00013922	1.133563526
ADD2	2.93E-05	-1.13289427	ST6GAL2	2.95E-05	1.133563526
LRRC71	3.04E-05	-1.12973393	FOXP2	3.39E-05	1.132247798
MEG3	3.27E-05	-1.123433941	GULP1	0.00066639	1.130271955
RP11-554I8.2	0.000265126	-1.120294234	CBLN1	0.0005547	1.130271955
HAPLN4	0.000220502	-1.120294234	ART3	3.33E-05	1.130271955
DNAH11	4.14E-05	-1.120294234	NEURL3	2.93E-05	1.130271955
UNC13A	2.96E-05	-1.120294234	KLF6	2.93E-05	1.128293401
SPRED3	2.93E-05	-1.117161344	TLR2	0.00015222	1.12763328
ATP2B3	2.93E-05	-1.117161344	ZC3H12A	7.24E-05	1.126972856
SYT5	2.93E-05	-1.117161344	CD69	0.00046195	1.12631213
NDUFA4L2	2.93E-05	-1.117161344	TMEM56	3.33E-05	1.12631213
PRKCA	2.93E-05	-1.114035243	ARSJ	3.08E-05	1.12498977
RASSF9	0.001249602	-1.110915901	RORB	0.00016685	1.122341408
CXorf27	2.99E-05	-1.10780329	CRYBA1	2.98E-05	1.121015401
LRRC46	2.98E-05	-1.10780329	DRD1	0.00192956	1.12035194
TTC40	2.93E-05	-1.10780329	GEM	0.00095782	1.12035194
TMCC2	2.93E-05	-1.10780329	RP11-792D21.2	0.00087605	1.117695043
LRRC4B	2.93E-05	-1.10780329	C2CD4B	0.03131526	1.113033665
DLG3	2.93E-05	-1.10780329	CYTL1	7.24E-05	1.110363243
ALPK1	0.000127607	-1.104697379	TMEM107	2.93E-05	1.110363243
HYDIN	4.98E-05	-1.104697379	ATF3	0.00114331	1.109694865

CTNNA2	2.96E-05	-1.104697379	ATP10A	0.00012761	1.109694865
SLC45A4	2.93E-05	-1.104697379	TDRD6	3.46E-05	1.109694865
C16orf46	2.93E-05	-1.104697379	SATB2	0.00474318	1.109026176
RP11-122F24.1	3.46E-05	-1.10159814	CNTN4	0.00405021	1.108357178
TDRD9	3.08E-05	-1.10159814	GRASP	3.22E-05	1.108357178
LINC00683	3.06E-05	-1.10159814	AQP4	5.25E-05	1.10701825
SLC26A10	2.93E-05	-1.10159814	C6orf141	0.00087605	1.105678078
GRIN1	2.93E-05	-1.10159814	RAB32	2.93E-05	1.105678078
CENPT	2.93E-05	-1.10159814	CXorf21	3.04E-05	1.10433666
GPR64	0.037571178	-1.098505545	C17orf96	0.0043847	1.103665483
KCNIP2	2.97E-05	-1.098505545	ZFP42	0.01430577	1.102993993
MCTP2	2.97E-05	-1.098505545	TRIM38	3.84E-05	1.102993993
TMEM120B	2.93E-05	-1.098505545	PDLIM3	3.54E-05	1.101650076
SLC22A7	0.000166853	-1.095419565	DOCK2	3.27E-05	1.101650076
PRKCE	2.93E-05	-1.095419565	EPHA3	0.00104668	1.100304906
PDZD4	2.93E-05	-1.095419565	SOX6	2.93E-05	1.100304906
EFR3B	2.93E-05	-1.095419565	ADAMTS9	7.80E-05	1.09963185
GIT1	2.93E-05	-1.095419565	GLP2R	0.0002205	1.098284796
KIT	3.62E-05	-1.092340172	HRH1	2.94E-05	1.097610797
SLC25A23	2.93E-05	-1.092340172	ST6GAL2	2.93E-05	1.096936483
FKBP5	0.037571178	-1.089267338	HK2	0.00052899	1.096261853
CSDC2	2.97E-05	-1.089267338	RCN1	3.54E-05	1.096261853
SLC25A23	2.93E-05	-1.089267338	MYO5C	3.03E-05	1.096261853
MEG3	2.93E-05	-1.089267338	TRIM22	3.11E-05	1.093560176
PLXNA4	3.19E-05	-1.086201035	ADAMTSL3	3.06E-05	1.092883966
SHANK1	2.98E-05	-1.086201035	AOAH	2.93E-05	1.091530593
ITGA4	2.93E-05	-1.086201035	MEPE	0.00104668	1.09085343
NEK2	0.000799754	-1.083141235	STXBP5-AS1	0.00034969	1.09085343
CLSTN2	2.97E-05	-1.083141235	L3MBTL4	2.93E-05	1.087462841
GABRQ	0.003447984	-1.080087911	TLR4	2.95E-05	1.086104371
PIH1D3	0.001365182	-1.080087911	MMP3	0.00060839	1.084744621
RESP18	0.000957819	-1.080087911	DRAXIN	0.00010746	1.084744621
KSR2	2.93E-05	-1.080087911	TRIM22	2.96E-05	1.084744621
SHANK2	2.93E-05	-1.077041036	CD33	6.32E-05	1.083383588
ESCO2	8.43E-05	-1.074000581	HRH1	3.84E-05	1.082021269
C9orf117	3.03E-05	-1.074000581	CPNE5	0.00034969	1.081339627
CTC-336P14.1	0.037571178	-1.070966521	MPP7	0.00749242	1.080657663
JPH1	2.93E-05	-1.070966521	ADRB2	2.93E-05	1.079292767
PRLR	0.008686065	-1.067938829	WEE1	2.98E-05	1.078609835
RSPH4A	3.39E-05	-1.064917477	CDNF	4.14E-05	1.077926579
MDGA1	3.08E-05	-1.064917477	PRKD3	3.27E-05	1.077242999
BTNL9	5.93E-05	-1.061902439	TNC	0.03131526	1.075874867
VSTM1	2.94E-05	-1.061902439	CD86	3.15E-05	1.075874867
SDK1	2.93E-05	-1.061902439	SPARCL1	2.94E-05	1.073134705
EHF	3.54E-05	-1.058893689	FAM83D	7.80E-05	1.071076162
RAB3D	2.93E-05	-1.058893689	DLEU1	4.31E-05	1.069702167
PRB4	0.000183144	-1.055891201	CDH13	0.00474318	1.069014678
TNFAIP8L3	2.94E-05	-1.055891201	RGS6	0.00020109	1.069014678
MCF2L	2.93E-05	-1.055891201	SIGLEC14	0.00292929	1.065572312
ADRA1A	0.000349687	-1.052894948	RGS6	0.00060839	1.065572312
FZD7	0.000116923	-1.052894948	TAGAP	3.13E-05	1.064882852
MSC	3.03E-05	-1.049904906	HSD17B11	2.93E-05	1.063502942
COL5A2	0.000666388	-1.046921047	LINC00324	2.94E-05	1.062121712
SUSD4	3.04E-05	-1.043943348	S100A9	0.04751155	1.060739158
PVALB	0.006954243	-1.040971781	TMEM132D	0.00598002	1.059355278
RSPO3	0.000152218	-1.040971781	LAPTM5	3.03E-05	1.059355278
INS-IGF2	0.000421702	-1.038006323	OR2L13	0.00405021	1.057970069
NEUROD2	2.95E-05	-1.038006323	PPM1B	2.93E-05	1.057970069
SYNPO	2.93E-05	-1.038006323	RRAD	0.00695424	1.057276965
SYNPO	2.93E-05	-1.038006323	LIFR	2.95E-05	1.055889758
INADL	2.93E-05	-1.038006323	AMIGO2	2.97E-05	1.055195654
CATSPERD	0.001625989	-1.035046947	BDKRB2	7.80E-05	1.048933645
PAGE4	0.000799754	-1.035046947	OTUD1	2.93E-05	1.048933645
PCDH20	0.000241964	-1.035046947	IL33	3.04E-05	1.047538389
SAMD14	2.93E-05	-1.035046947	SLC1A3	2.96E-05	1.047538389
KCTD13	2.93E-05	-1.035046947	KDM6A	2.93E-05	1.047538389
ZFHX2	2.93E-05	-1.035046947	CHRNA3	0.00104668	1.046840254
IQSEC3	2.93E-05	-1.03209363	FXYD6	3.15E-05	1.046840254
FOLR1	0.000290431	-1.029146346	SFMBT2	2.93E-05	1.046840254
SEZ6L	2.93E-05	-1.029146346	IL25	0.00026513	1.044044333

CAMK2B	2.97E-05	-1.02620507	ROPN1B	5.25E-05	1.04194383
TMEM2	2.93E-05	-1.02620507	GBP1	7.80E-05	1.041242982
HUNK	2.93E-05	-1.02620507	SLC44A3	4.73E-05	1.040541794
CCDC60	7.80E-05	-1.020340448	GCNT4	0.00104668	1.039138394
EPHA4	2.96E-05	-1.020340448	SCIN	0.00598002	1.038436182
STX1B	2.93E-05	-1.020340448	SLC7A11	2.93E-05	1.038436182
AC145110.1	0.000957819	-1.017417053	PRR16	2.93E-05	1.036327492
SYNE1	2.97E-05	-1.017417053	STXBP5-AS1	0.00177207	1.03562391
SH3RF1	2.96E-05	-1.017417053	TNFAIP8L2	2.95E-05	1.03562391
AC004540.5	2.93E-05	-1.017417053	NFKBIA	9.12E-05	1.034215715
NRP1	2.93E-05	-1.017417053	RIMS3	0.01160103	1.033511102
CTXN3	0.010802019	-1.01449957	PPAP2B	3.03E-05	1.033511102
SNAI3-AS1	2.94E-05	-1.01449957	DPP10	2.93E-05	1.033511102
NCDN	2.93E-05	-1.01449957	ZCCHC6	2.93E-05	1.033511102
JPH3	2.93E-05	-1.01449957	MB21D2	0.00038417	1.032100843
CACNA2D3	2.93E-05	-1.01449957	KLHL6	9.48E-05	1.031395196
TNFRSF25	2.98E-05	-1.011587974	CRLF1	4.51E-05	1.030689204
WASF1	2.93E-05	-1.011587974	ST7-OT4	2.93E-05	1.030689204
PPP1R9B	2.93E-05	-1.011587974	ARHGAP25	3.15E-05	1.029982866
FGF13	2.99E-05	-1.008682243	IL18	9.88E-05	1.029276182
DOCK4	2.93E-05	-1.008682243	C12orf75	2.93E-05	1.029276182
NEK10	2.93E-05	-1.005782353	VSTM2A	0.01244972	1.028569152
CCDC108	2.93E-05	-1.005782353	EDNRB	2.95E-05	1.028569152
AC145110.1	0.000265126	-1	PLD4	2.94E-05	1.028569152
HLA-DMB	4.31E-05	-1	S1PR3	0.00013922	1.027861775
WDR96	3.98E-05	-1	RP11-632F7.1	3.01E-05	1.025737561
NOV	3.84E-05	-1	GDPD2	4.31E-05	1.025028794
ADORA2A	2.93E-05	-1	LPAR5	3.11E-05	1.025028794
CES1	0.000957819	1	AC144521.1	0.00079975	1.024319679
			SELE	0.000220502	1.024319679
			GIMAP5	2.94E-05	1.024319679
			SORCS1	0.002698525	1.022900402
			GPR137C	3.04E-05	1.022900402
			TMOD3	0.001046679	1.020768865
			ALOX5	0.000349687	1.020768865
			CHD2	3.46E-05	1.019346089
			UTS2B	0.00072996	1.018634174
			RP11-145M9.4	2.93E-05	1.018634174
			CXCL3	0.017538628	1.017921908
			CDH7	0.0093476	1.017921908
			MCL1	3.19E-05	1.017921908
			ATP13A4	2.95E-05	1.017921908
			KLF11	2.93E-05	1.017921908
			MSR1	0.001365182	1.015069322
			C1orf101	2.97E-05	1.015069322
			PCDH18	0.000318489	1.014355293
			PCSK1	0.000265126	1.014355293
			ARHGAP15	0.000241964	1.013640911
			WBP2NL	5.93E-05	1.012926174
			RARB	6.32E-05	1.012211084
			SLC16A8	2.93E-05	1.010779839
			TTN	3.30E-05	1.010063683
			KDM6A	2.93E-05	1.009347172
			CPED1	3.54E-05	1.008630305
			HSD17B6	2.93E-05	1.008630305
			FCGR1A	7.24E-05	1.007913082
			ZFP37	2.93E-05	1.007913082
			PHTF2	3.13E-05	1.006477564
			HLA-DMB	0.001046679	1.005759269
			ADAM28	7.24E-05	1.005759269
			ABI3BP	0.000152218	1.003602237
			CFD	2.99E-05	1.003602237
			GPR125	5.93E-05	1.002162421
			FMN1	0.003177654	1.001441974
			CPB2	9.88E-05	1.001441974
			KCNQ1	6.75E-05	1.001441974
			RPL23	2.98E-05	1.000721167

Supplemental Table 2. Complete list of significantly upregulated KEGG pathways in hippocampal tissue from epileptic patients compared to controls.

KEGG Pathway	Adjusted p-value	Pathway Size	Number of Upregulated Genes in Pathway	Upregulated Genes in Pathway
Rheumatoid arthritis	1.30E-11	88	18	IL18,FOS,CD86,MMP3,HLA-DRB1,TNF,HLA-DRB5,CXCL1,IL1B,CCL2,TLR2,CXCL3,TNFRSF11A,HLA-DQA2,HLA-DMB,TLR4,HLA-DRA,CCL3
Leishmaniasis	8.05E-11	72	16	C3,NFKBIA,FOS,NCF2,HLA-DRB1,TNF,HLA-DRB5,FCGR1A,IL1B,TLR2,PTGS2,CYBB,HLA-DQA2,HLA-DMB,TLR4,HLA-DRA
Staphylococcus aureus infection	2.10E-06	87	13	C3,CFD,C1QB,HLA-DRB1,FPR1,HLA-DRB5,FCGR1A,C1QA,FCAR,HLA-DQA2,CFB,HLA-DMB,HLA-DRA
Phagosome	4.98E-06	147	16	C3,NCF2,THBS1,HLA-DRB1,MSR1,HLA-DRB5,FCGR1A,TLR2,OLR1,CLEC7A,FCAR,CYBB,HLA-DQA2,HLA-DMB,TLR4,HLA-DRA
Legionellosis	1.99E-05	57	10	C3,IL18,NFKBIA,CASP1,TNF,CXCL1,IL1B,TLR2,CXCL3,TLR4
IL-17 signaling pathway	3.48E-05	92	12	NFKBIA,FOS,IL25,MMP3,S100A9,TNF,FOSB,CXCL1,IL1B,CCL2,CXCL3,PTGS2
Inflammatory bowel disease	4.53E-05	62	10	IL18,HLA-DRB1,TNF,HLA-DRB5,IL1B,TLR2,HLA-DQA2,HLA-DMB,TLR4,HLA-DRA
Cytokine-cytokine receptor interaction	5.36E-05	292	21	IL18,CSF1R,TNFSF18,IL25,PPBP,CSF2RA,OSM,CCL8,IL33,TNF,CX3CR1,CXCL1,IL1B,CCL2,CXCL3,TNFRSF11A,LIFR,IL6ST,IL1RN,CCL3,TNFRSF10D
Graft-versus-host disease	6.37E-05	37	8	CD86,HLA-DRB1,TNF,HLA-DRB5,IL1B,HLA-DQA2,HLA-DMB,HLA-DRA
Viral protein interaction with cytokine and cytokine receptor	7.00E-05	98	12	IL18,CSF1R,PPBP,CCL8,TNF,CX3CR1,CXCL1,CCL2,CXCL3,IL6ST,CCL3,TNFRSF10D
Chagas disease	9.73E-05	101	12	C3,NFKBIA,FOS,C1QB,BDKRB2,TNF,IL1B,CCL2,TLR2,C1QA,TLR4,CCL3
NF-kappa B signaling pathway	0.00010836	102	12	NFKBIA,BCL2A1,TNF,CXCL1,IL1B,SYK,CXCL3,TNFRSF11A,GADD45B,PTGS2,TLR4,GADD45G
Type I diabetes mellitus	0.00012037	40	8	CD86,HLA-DRB1,TNF,HLA-DRB5,IL1B,HLA-DQA2,HLA-DMB,HLA-DRA
Osteoclast differentiation	0.00015921	125	13	NFKBIA,FOS,CSF1R,NCF2,TREM2,SOCS3,TNF,FOSB,FCGR1A,IL1B,SYK,TNFRSF11A,LILRB4
Influenza A	0.00017496	168	15	IL18,NFKBIA,CASP1,SOCS3,HLA-DRB1,IL33,TNF,HLA-DRB5,TLR7,IL1B,CCL2,HLA-DQA2,HLA-DMB,TLR4,HLA-DRA
Tuberculosis	0.0002923	175	15	C3,IL18,HLA-DRB1,TNF,HLA-DRB5,FCGR1A,IL1B,TLR2,SYK,CLEC7A,HLA-DQA2,HLA-DMB,TLR4,HLA-DRA,IRAK2
Hematopoietic cell lineage	0.00036089	95	11	CSF1R,CSF2RA,HLA-DRB1,CD33,TNF,HLA-DRB5,FCGR1A,IL1B,HLA-DQA2,HLA-DMB,HLA-DRA
Allograft rejection	0.00046537	34	7	CD86,HLA-DRB1,TNF,HLA-DRB5,HLA-DQA2,HLA-DMB,HLA-DRA
Neuroactive ligand-receptor interaction	0.00059061	339	21	C3,RXFP1,ADORA3,ADRB2,MCHR2,HRH1,F2R,TAC3,NPY,EDNRB,BDKRB2,FPR1,DRD1,NMU,S1PR3,HTR2A,GRIK3,UTS2B,HTR1D,CHRNA3,GLP2R
Malaria	0.00060235	49	8	IL18,THBS1,TNF,IL1B,CCL2,TLR2,SELE,TLR4
Asthma	0.00143096	27	6	HLA-DRB1,TNF,HLA-DRB5,HLA-DQA2,HLA-DMB,HLA-DRA
TNF signaling pathway	0.00179017	112	11	NFKBIA,FOS,MMP3,SOCS3,TNF,CXCL1,IL1B,CCL2,SELE,CXCL3,PTGS2

Pertussis	0.00235632	76	9	C3,FOS,C1QB,CASP1,TNF,IL1B,C1QA,TLR4,IRF8
Complement and coagulation cascades	0.00580725	85	9	C3,CFD,C1QB,F2R,VSIG4,BDKRB2,CPB2,C1QA,CFB
Toxoplasmosis	0.00777973	109	10	ALOX5,NFKBIA,HLA-DRB1,TNF,HLA-DRB5,TLR2,HLA-DQA2,HLA-DMB,TLR4,HLA-DRA
Systemic lupus erythematosus	0.00826235	132	11	C3,CD86,C1QB,HLA-DRB1,TNF,HLA-DRB5,FCGR1A,C1QA,HLA-DQA2,HLA-DMB,HLA-DRA
Transcriptional misregulation in cancer	0.01516712	191	13	CD86,CSF1R,BCL2A1,MMP3,NFKBIZ,MYC,KDM6A,NR4A3,MEF2C,SPINT1,FCGR1A,GADD45B,GADD45G
Pathogenic Escherichia coli infection	0.01597983	192	13	IL18,NFKBIA,FOS,NCKAP1L,MYO5C,SEC24D,CASP1,LPAR5,F2R,TNF,CLDN10,IL1B,TLR4
Toll-like receptor signaling pathway	0.02392381	102	9	NFKBIA,FOS,CD86,TNF,TLR7,IL1B,TLR2,TLR4,CCL3
Th17 cell differentiation	0.0277027	104	9	NFKBIA,FOS,HLA-DRB1,HLA-DRB5,IL1B,HLA-DQA2,IL6ST,HLA-DMB,HLA-DRA
NOD-like receptor signaling pathway	0.02825788	177	12	IL18,NFKBIA,CASP1,TNF,GBP1,CXCL1,IL1B,CCL2,CXCL3,CYBB,TLR4,PYDC2
Intestinal immune network for IgA production	0.02892439	45	6	CD86,HLA-DRB1,HLA-DRB5,HLA-DQA2,HLA-DMB,HLA-DRA
Autoimmune thyroid disease	0.04637981	49	6	CD86,HLA-DRB1,HLA-DRB5,HLA-DQA2,HLA-DMB,HLA-DRA

Supplemental Table 3. Complete list of significantly enriched GO Biological Processes in hippocampal tissue from epileptic patients compared to controls.

Downregulated Biological Processes	FDR	Upregulated Biological Process	FDR
cellular nitrogen compound biosynthetic process (GO:0044271)	1.65E-03	mRNA processing (GO:0006397)	1.76E-02
organonitrogen compound biosynthetic process (GO:1901566)	6.67E-03	RNA splicing (GO:0008380)	4.68E-02
macromolecule biosynthetic process (GO:0009059)	6.27E-03	modification-dependent macromolecule catabolic process (GO:0043632)	4.06E-02
cellular macromolecule biosynthetic process (GO:0034645)	1.15E-02	RNA processing (GO:0006396)	3.04E-02
catabolic process (GO:0009056)	4.20E-03	Unclassified (UNCLASSIFIED)	3.71E-04
cellular catabolic process (GO:0044248)	1.43E-02	nucleic acid metabolic process (GO:0090304)	1.82E-02
small molecule metabolic process (GO:0044281)	2.31E-02	nucleobase-containing compound metabolic process (GO:0006139)	4.44E-02
cellular biosynthetic process (GO:0044249)	1.08E-03	heterocycle metabolic process (GO:0046483)	2.91E-02
organic substance biosynthetic process (GO:1901576)	1.40E-03	cellular aromatic compound metabolic process (GO:0006725)	3.89E-02
organic substance catabolic process (GO:1901575)	4.69E-02	cellular nitrogen compound metabolic process (GO:0034641)	4.21E-02
biosynthetic process (GO:0009058)	2.27E-03	biological_process (GO:0008150)	3.69E-04
cellular nitrogen compound metabolic process (GO:0034641)	7.96E-04	cellular process (GO:0009987)	1.49E-05
nucleobase-containing compound metabolic process (GO:0006139)	1.17E-02	regulation of macromolecule metabolic process (GO:0060255)	3.24E-02
heterocycle metabolic process (GO:0046483)	1.23E-02	regulation of cellular metabolic process (GO:0031323)	4.21E-02
cellular aromatic compound metabolic process (GO:0006725)	1.38E-02	regulation of biological process (GO:0050789)	2.30E-07
organic cyclic compound metabolic process (GO:1901360)	1.91E-02	biological regulation (GO:0065007)	4.93E-09
primary metabolic process (GO:0044238)	1.29E-02	regulation of cellular process (GO:0050794)	4.01E-07
cellular metabolic process (GO:0044237)	1.16E-02	regulation of metabolic process (GO:0019222)	3.52E-03
metabolic process (GO:0008152)	1.18E-02	localization (GO:0051179)	1.14E-02
organic substance metabolic process (GO:0071704)	2.50E-02	regulation of gene expression (GO:0010468)	3.28E-03
regulation of biological process (GO:0050789)	2.49E-02	positive regulation of nitrogen compound metabolic process (GO:0051173)	1.76E-02
regulation of cellular process (GO:0050794)	2.23E-02	multicellular organismal process (GO:0032501)	1.81E-07
cell communication (GO:0007154)	3.80E-02	regulation of signal transduction (GO:0009966)	5.25E-03
signaling (GO:0023052)	1.53E-02	developmental process (GO:0032502)	1.01E-06
cellular component organization or biogenesis (GO:0071840)	1.70E-03	positive regulation of macromolecule metabolic process (GO:0010604)	1.20E-03
cellular component organization (GO:0016043)	3.58E-04	positive regulation of cellular metabolic process (GO:0031325)	1.10E-03
multicellular organismal process (GO:0032501)	1.38E-06	system process (GO:0003008)	3.89E-02
localization (GO:0051179)	4.93E-05	negative regulation of biological process (GO:0048519)	2.77E-07
cellular component biogenesis (GO:0044085)	4.06E-02	positive regulation of metabolic process (GO:0009893)	1.70E-04
regulation of signal transduction (GO:0009966)	1.61E-02	negative regulation of gene expression (GO:0010629)	3.63E-02
developmental process (GO:0032502)	7.14E-07	cellular developmental process (GO:0048869)	4.00E-04
anatomical structure development (GO:0048856)	1.28E-06	response to stimulus (GO:0050896)	3.25E-15
animal organ development (GO:0048513)	2.26E-03	anatomical structure development (GO:0048856)	3.12E-07

regulation of multicellular organismal process (GO:0051239)	8.91E-03	regulation of cell communication (GO:0010646)	3.87E-04
regulation of developmental process (GO:0050793)	1.61E-02	regulation of signaling (GO:0023051)	3.18E-04
regulation of molecular function (GO:0065009)	2.25E-03	positive regulation of cellular process (GO:0048522)	4.14E-08
cellular developmental process (GO:0048869)	1.81E-04	multicellular organism development (GO:0007275)	1.35E-06
cell differentiation (GO:0030154)	2.18E-04	cell differentiation (GO:0030154)	2.10E-04
multicellular organism development (GO:0007275)	2.51E-07	negative regulation of cellular process (GO:0048523)	5.67E-07
anatomical structure morphogenesis (GO:0009653)	1.21E-02	animal organ development (GO:0048513)	2.52E-04
cellular component assembly (GO:0022607)	5.54E-03	tissue development (GO:0009888)	2.59E-02
regulation of cell communication (GO:0010646)	7.52E-05	regulation of protein metabolic process (GO:0051246)	9.62E-04
regulation of signaling (GO:0023051)	5.56E-05	anatomical structure morphogenesis (GO:0009653)	6.98E-03
system development (GO:0048731)	8.41E-07	system development (GO:0048731)	1.32E-06
positive regulation of molecular function (GO:0044093)	1.63E-02	positive regulation of biological process (GO:0048518)	1.02E-11
tissue development (GO:0009888)	1.41E-02	cell development (GO:0048468)	2.51E-02
nervous system process (GO:0050877)	3.41E-02	cellular response to stimulus (GO:0051716)	5.46E-14
system process (GO:0003008)	2.04E-03	regulation of cellular protein metabolic process (GO:0032268)	6.52E-04
regulation of cell differentiation (GO:0045595)	1.41E-02	positive regulation of gene expression (GO:0010628)	1.11E-03
regulation of biological quality (GO:0065008)	7.32E-09	positive regulation of protein metabolic process (GO:0051247)	8.48E-03
regulation of localization (GO:0032879)	5.28E-07	positive regulation of signal transduction (GO:0009967)	1.43E-02
cell adhesion (GO:0007155)	4.87E-02	positive regulation of signaling (GO:0023056)	6.68E-03
cell-cell signaling (GO:0007267)	1.52E-02	negative regulation of signaling (GO:0023057)	2.80E-02
positive regulation of transport (GO:0051050)	4.62E-02	negative regulation of cell communication (GO:0010648)	2.76E-02
ion transport (GO:0006811)	3.54E-03	positive regulation of cell communication (GO:0010647)	6.38E-03
animal organ morphogenesis (GO:0009887)	1.60E-02	regulation of molecular function (GO:0065009)	1.40E-05
regulation of cellular component movement (GO:0051270)	5.56E-03	regulation of catalytic activity (GO:0050790)	3.85E-04
cytoskeleton organization (GO:0007010)	1.01E-03	regulation of biological quality (GO:0065008)	5.98E-08
ion transmembrane transport (GO:0034220)	1.13E-02	regulation of response to stimulus (GO:0048583)	2.06E-09
regulation of secretion (GO:0051046)	4.05E-02	response to chemical (GO:0042221)	5.90E-10
negative regulation of cellular component organization (GO:0051129)	2.88E-02	regulation of hydrolase activity (GO:0051336)	1.49E-02
negative regulation of cell differentiation (GO:0045596)	4.59E-02	positive regulation of cellular protein metabolic process (GO:0032270)	3.12E-03
cation transport (GO:0006812)	1.03E-02	intracellular signal transduction (GO:0035556)	2.66E-03
regulation of transport (GO:0051049)	7.92E-07	cell communication (GO:0007154)	1.01E-14
inorganic ion transmembrane transport (GO:0098660)	3.22E-02	signaling (GO:0023052)	6.60E-15
regulation of secretion by cell (GO:1903530)	4.08E-02	export from cell (GO:0140352)	3.90E-02
locomotion (GO:0040011)	5.21E-05	generation of neurons (GO:0048699)	1.30E-02
cell development (GO:0048468)	2.24E-06	regulation of protein modification process (GO:0031399)	4.88E-04
localization of cell (GO:0051674)	3.76E-04	response to stress (GO:0006950)	8.08E-09

cell motility (GO:0048870)	3.70E-04	neuron differentiation (GO:0030182)	3.00E-02
central nervous system development (GO:0007417)	9.09E-04	signal transduction (GO:0007165)	2.48E-14
taxis (GO:0042330)	4.10E-02	regulation of developmental process (GO:0050793)	1.22E-05
chemotaxis (GO:0006935)	4.04E-02	regulation of kinase activity (GO:0043549)	4.94E-02
nervous system development (GO:0007399)	8.99E-10	secretion by cell (GO:0032940)	4.44E-02
organelle assembly (GO:0070925)	3.53E-03	regulation of transferase activity (GO:0051338)	2.64E-02
movement of cell or subcellular component (GO:0006928)	1.06E-07	regulation of localization (GO:0032879)	5.18E-07
microtubule-based process (GO:0007017)	1.40E-03	nervous system development (GO:0007399)	1.20E-05
regulation of vesicle-mediated transport (GO:0060627)	2.34E-02	secretion (GO:0046903)	1.14E-02
divalent inorganic cation homeostasis (GO:0072507)	2.99E-02	homeostatic process (GO:0042592)	2.60E-04
head development (GO:0060322)	6.35E-04	cellular response to endogenous stimulus (GO:0071495)	6.69E-03
regulation of cell projection organization (GO:0031344)	4.22E-03	positive regulation of catalytic activity (GO:0043085)	7.12E-04
metal ion transport (GO:0030001)	4.14E-03	regulation of apoptotic process (GO:0042981)	5.05E-04
calcium ion homeostasis (GO:0055074)	3.23E-02	positive regulation of protein modification process (GO:0031401)	2.17E-03
brain development (GO:0007420)	6.35E-04	positive regulation of molecular function (GO:0044093)	3.24E-05
regulation of plasma membrane bounded cell projection organization (GO:0120035)	3.20E-03	response to endogenous stimulus (GO:0009719)	7.38E-04
regulation of cytoskeleton organization (GO:0051493)	6.47E-03	negative regulation of response to stimulus (GO:0048585)	2.89E-05
neurogenesis (GO:0022008)	1.42E-08	regulation of programmed cell death (GO:0043067)	2.55E-04
regulation of nervous system development (GO:0051960)	1.41E-02	neuron development (GO:0048666)	2.20E-02
cell morphogenesis (GO:0000902)	2.15E-04	regulation of intracellular signal transduction (GO:1902531)	3.44E-05
regulation of neurogenesis (GO:0050767)	3.64E-02	regulation of cell death (GO:0010941)	6.79E-05
regulation of anatomical structure size (GO:0090066)	2.54E-03	movement of cell or subcellular component (GO:0006928)	1.01E-04
synaptic signaling (GO:0099536)	7.06E-03	regulated exocytosis (GO:0045055)	4.59E-02
regulation of cellular component size (GO:0032535)	1.25E-02	positive regulation of response to stimulus (GO:0048584)	2.14E-07
generation of neurons (GO:0048699)	8.83E-10	regulation of phosphorus metabolic process (GO:0051174)	8.42E-06
regulation of supramolecular fiber organization (GO:1902903)	9.23E-03	regulation of phosphate metabolic process (GO:0019220)	8.38E-06
cell morphogenesis involved in differentiation (GO:0000904)	3.29E-04	regulation of transport (GO:0051049)	1.01E-05
trans-synaptic signaling (GO:0099537)	4.06E-03	response to nitrogen compound (GO:1901698)	2.06E-03
cell junction assembly (GO:0034329)	3.81E-02	neurogenesis (GO:0022008)	2.74E-04
axonogenesis (GO:0007409)	4.29E-03	response to abiotic stimulus (GO:0009628)	1.31E-03
cellular component morphogenesis (GO:0032989)	4.70E-05	regulation of protein transport (GO:0051223)	4.80E-02
neuron differentiation (GO:0030182)	5.26E-09	head development (GO:0060322)	1.16E-02
chemical synaptic transmission (GO:0007268)	2.67E-03	G protein-coupled receptor signaling pathway (GO:0007186)	2.55E-04
anterograde trans-synaptic signaling (GO:0098916)	2.64E-03	regulation of multicellular organismal process (GO:0051239)	8.82E-10
plasma membrane bounded cell projection assembly (GO:0120031)	1.07E-03	immune effector process (GO:0002252)	1.29E-03
regulation of blood circulation (GO:1903522)	1.99E-02	regulation of phosphorylation (GO:0042325)	1.21E-05

cilium organization (GO:0044782)	3.74E -03	regulation of ion transport (GO:0043269)	1.59E -02
cell projection assembly (GO:0030031)	5.98E -04	negative regulation of cell death (GO:0060548)	1.94E -03
neuron development (GO:0048666)	1.16E -07	negative regulation of apoptotic process (GO:0043066)	3.51E -03
regulation of heart contraction (GO:0008016)	4.30E -02	hematopoietic or lymphoid organ development (GO:0048534)	3.06E -02
axon development (GO:0061564)	1.39E -03	cell-cell signaling (GO:0007267)	6.08E -04
regulation of neuron projection development (GO:0010975)	8.54E -04	biological adhesion (GO:0022610)	1.81E -03
plasma membrane bounded cell projection organization (GO:0120036)	9.61E -11	regulation of multicellular organismal development (GO:2000026)	6.53E -05
heart morphogenesis (GO:0003007)	4.20E -02	localization of cell (GO:0051674)	3.88E -04
regulation of metal ion transport (GO:0010959)	2.04E -03	cell motility (GO:0048870)	3.86E -04
regulation of membrane potential (GO:0042391)	7.96E -04	cell surface receptor signaling pathway (GO:0007166)	3.57E -10
regulation of actin filament organization (GO:0110053)	1.59E -02	cell adhesion (GO:0007155)	1.69E -03
cell morphogenesis involved in neuron differentiation (GO:0048667)	4.03E -04	response to organonitrogen compound (GO:0010243)	1.03E -03
regulation of actin cytoskeleton organization (GO:0032956)	2.57E -03	positive regulation of hydrolase activity (GO:0051345)	5.53E -03
developmental maturation (GO:0021700)	3.75E -02	locomotion (GO:0040011)	4.12E -05
neuron projection guidance (GO:0097485)	1.41E -02	response to organic substance (GO:0010033)	5.99E -13
axon guidance (GO:0007411)	1.38E -02	cellular response to chemical stimulus (GO:0070887)	1.42E -12
cell part morphogenesis (GO:0032990)	3.99E -05	immune system development (GO:0002520)	1.53E -02
cilium assembly (GO:0060271)	2.06E -03	regulation of protein phosphorylation (GO:0001932)	8.77E -06
plasma membrane bounded cell projection morphogenesis (GO:0120039)	5.54E -05	positive regulation of transferase activity (GO:0051347)	1.12E -02
cell projection organization (GO:0030030)	7.77E -13	negative regulation of programmed cell death (GO:0043069)	1.49E -03
neuron projection morphogenesis (GO:0048812)	5.02E -05	brain development (GO:0007420)	4.84E -03
divalent inorganic cation transport (GO:0072511)	7.34E -03	blood vessel development (GO:0001568)	4.58E -02
regulation of ion transport (GO:0043269)	8.86E -08	adaptive immune response (GO:0002250)	1.72E -02
cell junction organization (GO:0034330)	3.71E -05	cellular response to organonitrogen compound (GO:0071417)	1.54E -02
regulation of actin filament-based process (GO:0032970)	3.25E -04	positive regulation of intracellular signal transduction (GO:1902533)	2.83E -04
neuron projection development (GO:0031175)	1.21E -07	immune system process (GO:0002376)	1.42E -13
cell projection morphogenesis (GO:0048858)	2.02E -05	cell population proliferation (GO:0008283)	3.84E -02
divalent metal ion transport (GO:0070838)	6.09E -03	immune response-regulating cell surface receptor signaling pathway (GO:0002768)	3.79E -02
regulation of exocytosis (GO:0017157)	4.02E -02	hemopoiesis (GO:0030097)	1.22E -02
regulation of transmembrane transport (GO:0034762)	4.14E -07	positive regulation of apoptotic process (GO:0043065)	1.58E -02
regulation of system process (GO:0044057)	1.29E -07	regulation of system process (GO:0044057)	1.20E -02
calcium ion transmembrane transport (GO:0070588)	2.46E -02	positive regulation of transport (GO:0051050)	3.27E -04
behavior (GO:0007610)	3.19E -08	cellular response to nitrogen compound (GO:1901699)	4.95E -03
negative regulation of cell projection organization (GO:0031345)	1.67E -02	tube development (GO:0035295)	7.54E -04
calcium ion transport (GO:0006816)	1.48E -03	regulation of cellular component movement (GO:0051270)	9.89E -05
regulation of synapse organization (GO:0050807)	4.36E -03	muscle structure development (GO:0061061)	3.22E -02

negative regulation of neuron projection development (GO:0010977)	4.09E-02	synaptic signaling (GO:0099536)	4.28E-02
microtubule-based movement (GO:0007018)	9.59E-06	cell migration (GO:0016477)	1.61E-04
regulation of ion transmembrane transport (GO:0034765)	2.08E-08	regulation of cell differentiation (GO:0045595)	1.41E-07
regulation of axonogenesis (GO:0050770)	1.21E-02	granulocyte activation (GO:0036230)	2.25E-02
neuron migration (GO:0001764)	3.24E-02	positive regulation of protein phosphorylation (GO:0001934)	7.63E-05
adult behavior (GO:0030534)	1.50E-02	interspecies interaction between organisms (GO:0044419)	3.22E-11
regulation of synapse structure or activity (GO:0050803)	5.56E-04	positive regulation of programmed cell death (GO:0043068)	9.08E-03
multicellular organismal signaling (GO:0035637)	1.42E-02	immune response-regulating signaling pathway (GO:0002764)	2.10E-02
synapse organization (GO:0050808)	2.58E-05	positive regulation of kinase activity (GO:0033674)	4.66E-03
regulation of cation transmembrane transport (GO:1904062)	1.71E-07	positive regulation of protein kinase activity (GO:0045860)	1.12E-02
synapse assembly (GO:0007416)	3.98E-02	cellular response to organic substance (GO:0071310)	3.08E-13
regulation of neurotransmitter transport (GO:0051588)	3.83E-02	central nervous system development (GO:0007417)	2.72E-05
regulation of potassium ion transport (GO:0043266)	3.82E-02	negative regulation of intracellular signal transduction (GO:1902532)	1.46E-02
cognition (GO:0050890)	3.30E-07	response to oxygen-containing compound (GO:1901700)	1.40E-08
regulation of nervous system process (GO:0031644)	1.47E-03	response to organic cyclic compound (GO:0014070)	1.03E-04
cytosolic calcium ion transport (GO:0060401)	2.35E-02	negative regulation of developmental process (GO:0051093)	9.97E-05
neuromuscular process (GO:0050905)	4.48E-03	regulation of cell motility (GO:2000145)	3.72E-05
limbic system development (GO:0021761)	4.27E-03	positive regulation of protein transport (GO:0051222)	4.94E-02
regulation of neurotransmitter secretion (GO:0046928)	2.00E-02	regulation of cell population proliferation (GO:0042127)	2.04E-09
regulation of transmembrane transporter activity (GO:0022898)	2.10E-08	positive regulation of phosphorylation (GO:0042327)	5.18E-06
action potential (GO:0001508)	9.13E-03	positive regulation of phosphorus metabolic process (GO:0010562)	1.86E-06
learning or memory (GO:0007611)	3.74E-08	positive regulation of phosphate metabolic process (GO:0045937)	1.84E-06
regulation of synaptic transmission, glutamatergic (GO:0051966)	4.10E-02	positive regulation of developmental process (GO:0051094)	3.33E-07
hippocampus development (GO:0021766)	1.68E-02	response to external stimulus (GO:0009605)	1.46E-15
regulation of ion transmembrane transporter activity (GO:0032412)	1.16E-08	regulation of metal ion transport (GO:0010959)	2.37E-02
regulation of transporter activity (GO:0032409)	2.01E-09	regulation of locomotion (GO:0040012)	8.88E-06
negative regulation of axonogenesis (GO:0050771)	3.38E-02	cell activation involved in immune response (GO:0002263)	1.05E-03
sperm motility (GO:0097722)	6.13E-03	cell-cell adhesion (GO:0098609)	4.75E-03
flagellated sperm motility (GO:0030317)	6.08E-03	response to drug (GO:0042493)	2.11E-02
positive regulation of synaptic transmission (GO:0050806)	1.67E-04	regulation of anatomical structure size (GO:0090066)	3.30E-03
dendrite development (GO:0016358)	9.73E-04	leukocyte activation involved in immune response (GO:0002366)	9.61E-04
regulation of signaling receptor activity (GO:0010469)	2.68E-06	immune response (GO:0006955)	1.14E-12
learning (GO:0007612)	1.84E-05	positive regulation of cell population proliferation (GO:0008284)	1.46E-05
regulation of trans-synaptic signaling (GO:0099177)	1.01E-16	positive regulation of protein serine/threonine kinase activity (GO:0071902)	4.90E-02
modulation of chemical synaptic transmission (GO:0050804)	1.87E-16	regulation of cell migration (GO:0030334)	1.89E-05
postsynapse organization (GO:0099173)	4.22E-03	activation of immune response (GO:0002253)	2.95E-03

memory (GO:0007613)	9.23E-05	regulation of immune response (GO:0050776)	5.73E-07
associative learning (GO:0008306)	3.77E-03	negative regulation of multicellular organismal process (GO:0051241)	2.00E-06
protein localization to synapse (GO:0035418)	2.00E-02	multicellular organismal homeostasis (GO:0048871)	2.45E-02
cilium-dependent cell motility (GO:0060285)	1.78E-04	circulatory system process (GO:0003013)	3.55E-03
cilium or flagellum-dependent cell motility (GO:0001539)	1.75E-04	myeloid cell activation involved in immune response (GO:0002275)	2.50E-03
nerve development (GO:0021675)	3.11E-03	positive regulation of cell death (GO:0010942)	4.95E-04
cilium movement involved in cell motility (GO:0060294)	4.09E-04	regulation of secretion by cell (GO:1903530)	4.93E-04
long-term synaptic potentiation (GO:0060291)	4.49E-02	regulation of response to stress (GO:0080134)	1.27E-10
visual learning (GO:0008542)	4.18E-02	forebrain development (GO:0030900)	1.14E-02
regulation of cation channel activity (GO:2001257)	7.60E-09	positive regulation of immune response (GO:0050778)	8.03E-05
regulation of neuron migration (GO:2001222)	3.99E-02	regulation of immune system process (GO:0002682)	4.48E-12
cellular response to vascular endothelial growth factor stimulus (GO:0035924)	3.76E-02	negative regulation of cell differentiation (GO:0045596)	2.83E-04
microtubule bundle formation (GO:0001578)	2.27E-05	regulation of MAPK cascade (GO:0043408)	9.32E-05
intraspecies interaction between organisms (GO:0051703)	1.24E-02	positive regulation of multicellular organismal process (GO:0051240)	4.59E-10
social behavior (GO:0035176)	1.23E-02	muscle organ development (GO:0007517)	4.99E-02
neuromuscular junction development (GO:0007528)	3.24E-02	positive regulation of MAPK cascade (GO:0043410)	1.26E-03
dendrite morphogenesis (GO:0048813)	4.14E-03	regulation of epithelial cell proliferation (GO:0050678)	1.75E-02
cilium movement (GO:0003341)	4.05E-08	lymphocyte activation (GO:0046649)	8.36E-03
regulation of synaptic plasticity (GO:0048167)	5.03E-11	negative regulation of inflammatory response (GO:0050728)	2.24E-02
modulation of excitatory postsynaptic potential (GO:0098815)	6.76E-03	cell activation (GO:0001775)	7.84E-08
extracellular transport (GO:0006858)	1.71E-02	striated muscle tissue development (GO:0014706)	4.07E-02
epithelial cilium movement involved in extracellular fluid movement (GO:0003351)	1.38E-02	positive regulation of secretion by cell (GO:1903532)	1.82E-02
semaphorin-plexin signaling pathway (GO:0071526)	3.90E-03	cellular response to organic cyclic compound (GO:0071407)	6.85E-04
ionotropic glutamate receptor signaling pathway (GO:0035235)	4.25E-02	regulation of trans-synaptic signaling (GO:0099177)	2.65E-03
positive regulation of synaptic transmission, glutamatergic (GO:0051968)	1.26E-02	modulation of chemical synaptic transmission (GO:0050804)	2.59E-03
axoneme assembly (GO:0035082)	8.71E-07	leukocyte activation (GO:0045321)	1.96E-07
bone resorption (GO:0045453)	3.96E-02	regulation of peptide secretion (GO:0002791)	5.22E-03
axonemal dynein complex assembly (GO:0070286)	3.16E-03	negative regulation of immune response (GO:0050777)	6.98E-03
regulation of neurotransmitter receptor activity (GO:0099601)	3.77E-08	vascular process in circulatory system (GO:0003018)	3.00E-02
neuron cellular homeostasis (GO:0070050)	1.04E-02	positive regulation of cell differentiation (GO:0045597)	6.51E-07
dendritic spine development (GO:0060996)	9.19E-03	negative regulation of transport (GO:0051051)	1.05E-03
regulation of NMDA receptor activity (GO:2000310)	6.06E-04	defense response to other organism (GO:0098542)	1.01E-08
exploration behavior (GO:0035640)	7.11E-03	telencephalon development (GO:0021537)	2.76E-02
regulation of Arp2/3 complex-mediated actin nucleation (GO:0034315)	2.47E-02	regulation of secretion (GO:0051046)	8.87E-06
innervation (GO:0060384)	6.30E-03	positive regulation of MAP kinase activity (GO:0043406)	3.68E-02
vocalization behavior (GO:0071625)	2.15E-02	negative regulation of defense response (GO:0031348)	1.61E-03

positive regulation of excitatory postsynaptic potential (GO:2000463)	1.14E-03	negative regulation of response to external stimulus (GO:0032102)	7.29E-05
vascular endothelial growth factor signaling pathway (GO:0038084)	4.73E-03	regulation of cysteine-type endopeptidase activity involved in apoptotic process (GO:0043281)	4.97E-02
regulation of AMPA receptor activity (GO:2000311)	8.60E-04	positive regulation of immune system process (GO:0002684)	6.14E-10
regulation of neuronal synaptic plasticity (GO:0048168)	4.59E-08	cellular response to oxygen-containing compound (GO:1901701)	3.92E-10
long-term synaptic depression (GO:0060292)	1.61E-02	myeloid leukocyte activation (GO:0002274)	2.20E-05
postsynapse assembly (GO:0099068)	1.60E-02	leukocyte differentiation (GO:0002521)	2.76E-03
regulation of long-term neuronal synaptic plasticity (GO:0048169)	7.51E-04	negative regulation of cell population proliferation (GO:0008285)	1.66E-06
dentate gyrus development (GO:0021542)	1.45E-02	blood circulation (GO:0008015)	7.94E-04
postsynaptic specialization organization (GO:0099084)	1.44E-02	humoral immune response (GO:0006959)	1.16E-03
dendritic spine morphogenesis (GO:0060997)	1.43E-02	activation of protein kinase activity (GO:0032147)	3.67E-03
postsynaptic density organization (GO:0097106)	1.43E-02	response to external biotic stimulus (GO:0043207)	1.23E-14
calcium ion-regulated exocytosis of neurotransmitter (GO:0048791)	1.26E-02	response to other organism (GO:0051707)	1.27E-14
outer dynein arm assembly (GO:0036158)	2.24E-03	cellular response to tumor necrosis factor (GO:0071356)	1.79E-02
facial nerve morphogenesis (GO:0021610)	4.94E-02	response to biotic stimulus (GO:0009607)	5.53E-15
cranial nerve structural organization (GO:0021604)	4.92E-02	regulation of protein secretion (GO:0050708)	2.17E-03
facial nerve development (GO:0021561)	4.90E-02	positive regulation of lymphocyte activation (GO:0051251)	9.73E-04
positive regulation of synaptic plasticity (GO:0031915)	4.88E-02	negative regulation of immune system process (GO:0002683)	8.84E-06
endothelial cell chemotaxis (GO:0035767)	4.86E-02	learning or memory (GO:0007611)	1.14E-02
negative regulation of collateral sprouting (GO:0048671)	4.09E-02	regulation of neuron death (GO:1901214)	3.11E-03
facial nerve structural organization (GO:0021612)	3.50E-02	response to cytokine (GO:0034097)	8.13E-12
regulation of synapse structural plasticity (GO:0051823)	3.48E-02	response to lipid (GO:0033993)	1.25E-08
modification of synaptic structure (GO:0099563)	3.47E-02	behavior (GO:0007610)	6.66E-06
postsynaptic specialization assembly (GO:0098698)	2.78E-02	regulation of lymphocyte activation (GO:0051249)	1.35E-05
postsynaptic density assembly (GO:0097107)	2.77E-02	taxis (GO:0042330)	7.81E-06
maintenance of postsynaptic density structure (GO:0099562)	2.16E-02	response to tumor necrosis factor (GO:0034612)	5.52E-03
maintenance of postsynaptic specialization structure (GO:0098880)	2.15E-02	chemotaxis (GO:0006935)	7.38E-06
axonogenesis involved in innervation (GO:0060385)	1.63E-02	regulation of immune effector process (GO:0002697)	4.16E-05
modification of postsynaptic structure (GO:0099010)	1.62E-02	defense response (GO:0006952)	6.94E-16
synaptic growth at neuromuscular junction (GO:0051124)	1.28E-02	cognition (GO:0050890)	2.06E-03
axonemal central apparatus assembly (GO:1904158)	1.27E-02	positive regulation of secretion (GO:0051047)	8.57E-04
		regulation of cell junction assembly (GO:1901888)	2.89E-02
		regulation of cell activation (GO:0050865)	2.14E-07
		innate immune response (GO:0045087)	1.38E-09
		regulation of response to external stimulus (GO:0032101)	4.54E-14
		regulation of neuron apoptotic process (GO:0043523)	1.75E-02
		response to mechanical stimulus (GO:0009612)	1.75E-02

		antigen processing and presentation of exogenous peptide antigen (GO:0002478)	4.13E-02
		negative regulation of cell adhesion (GO:0007162)	2.61E-03
		regulation of I-kappaB kinase/NF-kappaB signaling (GO:0043122)	6.41E-03
		regulation of DNA-binding transcription factor activity (GO:0051090)	3.99E-05
		positive regulation of cell motility (GO:2000147)	2.19E-06
		cellular response to cytokine stimulus (GO:0071345)	1.12E-12
		regulation of cell adhesion (GO:0030155)	1.52E-08
		positive regulation of cellular component movement (GO:0051272)	1.18E-06
		response to bacterium (GO:0009617)	7.63E-09
		positive regulation of cell migration (GO:0030335)	2.96E-06
		negative regulation of cell activation (GO:0050866)	1.43E-02
		negative regulation of cell-cell adhesion (GO:0022408)	1.98E-02
		positive regulation of locomotion (GO:0040017)	4.12E-07
		regulation of leukocyte activation (GO:0002694)	4.60E-08
		negative regulation of neuron apoptotic process (GO:0043524)	4.92E-02
		positive regulation of cell activation (GO:0050867)	1.12E-05
		G protein-coupled receptor signaling pathway, coupled to cyclic nucleotide second messenger (GO:0007187)	2.32E-03
		cellular response to lipid (GO:0071396)	1.29E-06
		regulation of hemopoiesis (GO:1903706)	2.12E-05
		positive regulation of leukocyte activation (GO:0002696)	7.62E-06
		regulation of lymphocyte differentiation (GO:0045619)	1.43E-02
		regulation of defense response (GO:0031347)	1.42E-12
		regulation of peptidyl-tyrosine phosphorylation (GO:0050730)	1.06E-03
		positive regulation of DNA-binding transcription factor activity (GO:0051091)	6.32E-04
		regulation of response to wounding (GO:1903034)	2.23E-02
		positive regulation of peptide secretion (GO:0002793)	7.94E-03
		positive regulation of ERK1 and ERK2 cascade (GO:0070374)	4.56E-03
		regulation of blood pressure (GO:0008217)	7.65E-03
		regulation of leukocyte differentiation (GO:1902105)	3.15E-04
		positive regulation of cytokine production (GO:0001819)	6.70E-07
		regulation of myeloid cell differentiation (GO:0045637)	1.29E-03
		negative regulation of leukocyte activation (GO:0002695)	6.29E-03
		myeloid cell differentiation (GO:0030099)	1.24E-03
		regulation of production of molecular mediator of immune response (GO:0002700)	1.77E-02
		positive regulation of cell adhesion (GO:0045785)	8.82E-07
		response to temperature stimulus (GO:0009266)	9.42E-03

		regulation of osteoblast differentiation (GO:0045667)	4.85E-02
		regulation of myeloid leukocyte differentiation (GO:0002761)	4.84E-02
		regulation of cytokine-mediated signaling pathway (GO:0001959)	8.63E-03
		skeletal muscle organ development (GO:0060538)	2.52E-02
		regulation of cytokine production (GO:0001817)	5.04E-13
		regulation of ERK1 and ERK2 cascade (GO:0070372)	4.42E-05
		renal system process (GO:0003014)	4.08E-02
		cellular response to interleukin-1 (GO:0071347)	3.92E-03
		leukocyte migration (GO:0050900)	1.19E-06
		cytokine-mediated signaling pathway (GO:0019221)	1.20E-12
		positive regulation of immune effector process (GO:0002699)	6.99E-04
		regulation of wound healing (GO:0061041)	2.12E-02
		cytokine production (GO:0001816)	6.38E-03
		regulation of cell-cell adhesion (GO:0022407)	7.94E-08
		glial cell development (GO:0021782)	3.60E-02
		response to interleukin-1 (GO:0070555)	1.07E-03
		negative regulation of lymphocyte activation (GO:0051250)	5.83E-03
		icosanoid metabolic process (GO:0006690)	3.42E-02
		positive regulation of protein secretion (GO:0050714)	3.05E-03
		positive regulation of response to external stimulus (GO:0032103)	8.79E-10
		regulation of chemotaxis (GO:0050920)	2.52E-04
		positive regulation of peptidyl-tyrosine phosphorylation (GO:0050731)	7.82E-04
		glial cell differentiation (GO:0010001)	1.39E-03
		skeletal muscle tissue development (GO:0007519)	1.58E-02
		regulation of leukocyte chemotaxis (GO:0002688)	1.58E-02
		regulation of response to cytokine stimulus (GO:0060759)	1.23E-03
		regulation of gliogenesis (GO:0014013)	4.91E-02
		negative regulation of transmembrane transport (GO:0034763)	1.45E-02
		regulation of T cell differentiation (GO:0045580)	3.59E-03
		regulation of mononuclear cell proliferation (GO:0032944)	8.38E-05
		regulation of T cell activation (GO:0050863)	4.46E-07
		regulation of lymphocyte proliferation (GO:0050670)	7.62E-05
		negative regulation of leukocyte cell-cell adhesion (GO:1903038)	6.37E-03
		regulation of tube size (GO:0035150)	3.31E-03
		regulation of blood vessel diameter (GO:0097746)	3.15E-03
		regulation of tube diameter (GO:0035296)	3.14E-03

		negative regulation of cytokine production (GO:0001818)	2.28E-06
		positive regulation of leukocyte differentiation (GO:1902107)	1.57E-03
		positive regulation of hemopoiesis (GO:1903708)	1.57E-03
		gliogenesis (GO:0042063)	3.10E-05
		regulation of adaptive immune response based on somatic recombination of immune receptors built from immunoglobulin superfamily domains (GO:0002822)	1.42E-03
		negative regulation of immune effector process (GO:0002698)	5.22E-03
		positive regulation of defense response (GO:0031349)	2.60E-08
		regulation of reactive oxygen species metabolic process (GO:2000377)	1.94E-04
		positive regulation of cell-cell adhesion (GO:0022409)	1.64E-06
		positive regulation of T cell activation (GO:0050870)	4.78E-05
		positive regulation of adaptive immune response (GO:0002821)	1.82E-02
		phospholipase C-activating G protein-coupled receptor signaling pathway (GO:0007200)	1.68E-02
		toll-like receptor signaling pathway (GO:0002224)	1.68E-02
		regulation of smooth muscle cell proliferation (GO:0048660)	1.98E-03
		regulation of leukocyte proliferation (GO:0070663)	3.62E-06
		regulation of fat cell differentiation (GO:0045598)	1.89E-03
		cell chemotaxis (GO:0060326)	2.86E-05
		myeloid leukocyte differentiation (GO:0002573)	3.56E-03
		negative regulation of T cell activation (GO:0050868)	3.55E-03
		regulation of adaptive immune response (GO:0002819)	2.28E-04
		regulation of inflammatory response (GO:0050727)	1.11E-14
		regulation of leukocyte cell-cell adhesion (GO:1903037)	1.54E-08
		regulation of leukocyte migration (GO:0002685)	1.17E-05
		regulation of lipid localization (GO:1905952)	3.99E-04
		positive regulation of chemotaxis (GO:0050921)	8.09E-04
		positive regulation of NF-kappaB transcription factor activity (GO:0051092)	1.84E-04
		positive regulation of mononuclear cell proliferation (GO:0032946)	6.45E-04
		positive regulation of leukocyte proliferation (GO:0070665)	3.06E-04
		negative regulation of leukocyte proliferation (GO:0070664)	1.14E-02
		positive regulation of fat cell differentiation (GO:0045600)	4.92E-02
		positive regulation of lymphocyte proliferation (GO:0050671)	6.13E-04
		negative regulation of T cell proliferation (GO:0042130)	4.91E-02
		regulation of T cell proliferation (GO:0042129)	6.77E-05
		positive regulation of lymphocyte differentiation (GO:0045621)	5.22E-03
		positive regulation of response to wounding (GO:1903036)	4.64E-02

		chemokine-mediated signaling pathway (GO:0070098)	2.12E-02
		positive regulation of leukocyte cell-cell adhesion (GO:1903039)	5.50E-07
		negative regulation of NF-kappaB transcription factor activity (GO:0032088)	8.84E-03
		inflammatory response (GO:0006954)	6.90E-16
		regulation of glucose transmembrane transport (GO:0010827)	1.87E-02
		regulation of response to oxidative stress (GO:1902882)	8.33E-03
		negative regulation of synaptic transmission (GO:0050805)	3.89E-02
		positive regulation of lipid localization (GO:1905954)	3.56E-03
		response to molecule of bacterial origin (GO:0002237)	1.97E-11
		regulation of synaptic transmission, glutamatergic (GO:0051966)	1.43E-02
		regulation of tumor necrosis factor production (GO:0032680)	4.88E-05
		mononuclear cell proliferation (GO:0032943)	6.00E-03
		positive regulation of reactive oxygen species metabolic process (GO:2000379)	1.17E-03
		positive regulation of T cell proliferation (GO:0042102)	1.16E-03
		response to lipopolysaccharide (GO:0032496)	5.49E-12
		regulation of tyrosine phosphorylation of STAT protein (GO:0042509)	5.26E-03
		lymphocyte proliferation (GO:0046651)	5.24E-03
		regulation of cytokine production involved in immune response (GO:0002718)	2.28E-03
		positive regulation of T cell differentiation (GO:0045582)	2.15E-03
		cellular response to chemokine (GO:1990869)	2.14E-03
		response to chemokine (GO:1990868)	2.13E-03
		regulation of tumor necrosis factor superfamily cytokine production (GO:1903555)	1.38E-05
		positive regulation of leukocyte migration (GO:0002687)	3.27E-05
		negative regulation of chemotaxis (GO:0050922)	2.60E-02
		response to fungus (GO:0009620)	2.59E-02
		positive regulation of cytokine production involved in immune response (GO:0002720)	2.58E-02
		regulation of cellular response to oxidative stress (GO:1900407)	4.59E-03
		response to interferon-gamma (GO:0034341)	3.92E-07
		leukocyte chemotaxis (GO:0030595)	1.21E-05
		regulation of oxidative stress-induced cell death (GO:1903201)	1.08E-02
		regulation of reactive oxygen species biosynthetic process (GO:1903426)	8.29E-04
		positive regulation of smooth muscle cell proliferation (GO:0048661)	1.87E-03
		positive regulation of wound healing (GO:0090303)	2.24E-02
		positive regulation of tumor necrosis factor production (GO:0032760)	1.61E-03
		leukocyte proliferation (GO:0070661)	6.29E-04
		myeloid leukocyte migration (GO:0097529)	1.65E-05

		regulation of phagocytosis (GO:0050764)	5.89E-04
		positive regulation of interleukin-1 production (GO:0032732)	7.90E-03
		positive regulation of interleukin-6 production (GO:0032755)	5.42E-04
		granulocyte migration (GO:0097530)	2.27E-04
		positive regulation of interleukin-1 beta production (GO:0032731)	1.81E-02
		neutrophil migration (GO:1990266)	5.00E-04
		positive regulation of gliogenesis (GO:0014015)	7.28E-03
		regulation of interleukin-6 production (GO:0032675)	1.81E-06
		regulation of mononuclear cell migration (GO:0071675)	1.70E-02
		regulation of cytokine secretion (GO:0050707)	1.69E-02
		regulation of alpha-beta T cell activation (GO:0046634)	1.81E-04
		cellular response to biotic stimulus (GO:0071216)	2.99E-10
		positive regulation of tumor necrosis factor superfamily cytokine production (GO:1903557)	4.26E-04
		regulation of interleukin-8 production (GO:0032677)	9.75E-04
		positive regulation of tyrosine phosphorylation of STAT protein (GO:0042531)	2.31E-03
		negative regulation of endothelial cell proliferation (GO:0001937)	3.89E-02
		osteoclast differentiation (GO:0030316)	3.88E-02
		cellular response to interferon-gamma (GO:0071346)	7.13E-08
		cellular response to molecule of bacterial origin (GO:0071219)	1.21E-09
		regulation of interleukin-12 production (GO:0032655)	5.28E-03
		cellular response to lipopolysaccharide (GO:0071222)	5.76E-10
		interferon-gamma-mediated signaling pathway (GO:0060333)	5.97E-04
		neutrophil chemotaxis (GO:0030593)	2.28E-04
		regulation of NIK/NF-kappaB signaling (GO:1901222)	3.10E-05
		positive regulation of reactive oxygen species biosynthetic process (GO:1903428)	4.09E-03
		positive regulation of NIK/NF-kappaB signaling (GO:1901224)	1.55E-03
		positive regulation of phagocytosis (GO:0050766)	1.54E-03
		granulocyte chemotaxis (GO:0071621)	7.65E-05
		leukocyte cell-cell adhesion (GO:0007159)	3.71E-03
		regulation of interleukin-1 production (GO:0032652)	9.53E-06
		positive regulation of interleukin-12 production (GO:0032735)	2.75E-02
		regulation of p38MAPK cascade (GO:1900744)	2.74E-02
		regulation of interferon-gamma production (GO:0032649)	3.13E-06
		regulation of CD4-positive, alpha-beta T cell activation (GO:2000514)	1.30E-03
		negative regulation of blood vessel diameter (GO:0097756)	2.52E-02
		positive regulation of interferon-gamma production (GO:0032729)	4.10E-04

		regulation of lipid storage (GO:0010883)	2.60E-03
		regulation of interleukin-1 beta production (GO:0032651)	1.20E-05
		positive regulation of interleukin-8 production (GO:0032757)	8.22E-04
		regulation of T-helper cell differentiation (GO:0045622)	1.88E-02
		vasodilation (GO:0042311)	1.87E-02
		negative regulation of interleukin-1 production (GO:0032692)	1.73E-02
		regulation of fatty acid transport (GO:2000191)	1.72E-02
		regulation of nitric oxide biosynthetic process (GO:0045428)	2.15E-04
		positive regulation of alpha-beta T cell activation (GO:0046635)	2.14E-04
		regulation of interleukin-10 production (GO:0032653)	6.16E-04
		purinergic nucleotide receptor signaling pathway (GO:0035590)	1.75E-03
		detection of biotic stimulus (GO:0009595)	1.58E-02
		MyD88-dependent toll-like receptor signaling pathway (GO:0002755)	1.57E-02
		negative regulation of interleukin-6 production (GO:0032715)	1.57E-03
		positive regulation of nitric oxide metabolic process (GO:1904407)	1.42E-03
		negative regulation of cell junction assembly (GO:1901889)	1.43E-02
		positive regulation of calcium-mediated signaling (GO:0050850)	1.43E-02
		skeletal muscle cell differentiation (GO:0035914)	4.41E-04
		positive regulation of nitric oxide biosynthetic process (GO:0045429)	1.28E-03
		regulation of cytokine production involved in inflammatory response (GO:1900015)	3.96E-04
		astrocyte differentiation (GO:0048708)	1.22E-04
		regulation of macrophage activation (GO:0043030)	1.21E-04
		regulation of macrophage migration (GO:1905521)	1.16E-03
		positive regulation of interleukin-10 production (GO:0032733)	3.30E-03
		protein kinase B signaling (GO:0043491)	3.29E-03
		regulation of chemokine production (GO:0032642)	2.11E-07
		positive regulation of inflammatory response (GO:0050729)	5.94E-13
		regulation of CD4-positive, alpha-beta T cell differentiation (GO:0043370)	2.83E-04
		leukotriene metabolic process (GO:0006691)	2.95E-03
		regulation of icosanoid secretion (GO:0032303)	3.27E-02
		regulation of alpha-beta T cell differentiation (GO:0046637)	6.29E-06
		negative regulation of interferon-gamma production (GO:0032689)	8.08E-04
		negative regulation of interleukin-1 beta production (GO:0032691)	8.71E-03
		positive regulation of interleukin-2 production (GO:0032743)	2.28E-03
		positive regulation of cytokine production involved in inflammatory response (GO:1900017)	2.89E-02
		astrocyte development (GO:0014002)	6.32E-04

		positive regulation of p38MAPK cascade (GO:1900745)	7.63E-03
		neuroinflammatory response (GO:0150076)	1.76E-04
		positive regulation of T-helper cell differentiation (GO:0045624)	2.54E-02
		detection of external biotic stimulus (GO:0098581)	2.53E-02
		positive regulation of alpha-beta T cell differentiation (GO:0046638)	3.44E-05
		macrophage activation (GO:0042116)	2.28E-06
		subpallium development (GO:0021544)	5.82E-03
		regulation of nitric-oxide synthase biosynthetic process (GO:0051769)	2.21E-02
		glial cell activation (GO:0061900)	1.13E-04
		positive regulation of lipid storage (GO:0010884)	5.80E-03
		regulation of acute inflammatory response (GO:0002673)	7.05E-06
		positive regulation of CD4-positive, alpha-beta T cell activation (GO:2000516)	3.70E-04
		positive regulation of interleukin-4 production (GO:0032753)	5.07E-03
		production of molecular mediator involved in inflammatory response (GO:0002532)	1.91E-02
		positive regulation of chemokine production (GO:0032722)	3.42E-07
		macrophage migration (GO:1905517)	1.90E-02
		astrocyte activation (GO:0048143)	1.90E-02
		regulation of interleukin-2 production (GO:0032663)	7.57E-08
		leukocyte activation involved in inflammatory response (GO:0002269)	2.76E-04
		microglial cell activation (GO:0001774)	2.74E-04
		positive regulation of oxidative stress-induced cell death (GO:1903209)	1.69E-02
		negative regulation of glucose transmembrane transport (GO:0010829)	1.68E-02
		eosinophil migration (GO:0072677)	1.68E-02
		regulation of interleukin-4 production (GO:0032673)	2.38E-04
		temperature homeostasis (GO:0001659)	2.05E-04
		lipopolysaccharide-mediated signaling pathway (GO:0031663)	4.42E-05
		detection of other organism (GO:0098543)	1.45E-02
		eosinophil chemotaxis (GO:0048245)	1.45E-02
		regulation of glial cell migration (GO:1903975)	1.45E-02
		positive regulation of CD4-positive, alpha-beta T cell differentiation (GO:0043372)	1.74E-04
		regulation of neuroinflammatory response (GO:0150077)	3.76E-05
		positive regulation of nitric-oxide synthase biosynthetic process (GO:0051770)	1.22E-02
		negative regulation of interleukin-2 production (GO:0032703)	1.19E-04
		striatum development (GO:0021756)	2.23E-03
		macrophage activation involved in immune response (GO:0002281)	1.02E-02
		regulation of granulocyte macrophage colony-stimulating factor production (GO:0032645)	1.02E-02

		positive regulation of macrophage migration (GO:1905523)	9.87E-05
		cranial nerve structural organization (GO:0021604)	4.59E-02
		negative regulation of sprouting angiogenesis (GO:1903671)	4.58E-02
		positive regulation of macrophage activation (GO:0043032)	8.08E-05
		positive regulation of monocyte chemotactic protein-1 production (GO:0071639)	3.79E-02
		chemokine production (GO:0032602)	3.79E-02
		complement activation, alternative pathway (GO:0006957)	6.78E-03
		positive regulation of acute inflammatory response (GO:0002675)	1.13E-06
		regulation of apoptotic cell clearance (GO:2000425)	3.03E-02
		diadenosine polyphosphate metabolic process (GO:0015959)	3.02E-02
		positive regulation of glial cell migration (GO:1903977)	4.33E-03
		positive regulation of apoptotic cell clearance (GO:2000427)	2.41E-02
		MHC protein complex assembly (GO:0002396)	1.86E-02
		oncostatin-M-mediated signaling pathway (GO:0038165)	1.85E-02
		regulation of heat generation (GO:0031650)	6.96E-05
		positive regulation of neuroinflammatory response (GO:0150078)	6.91E-05
		cell junction disassembly (GO:0150146)	2.56E-03
		positive regulation of heat generation (GO:0031652)	3.69E-04
		peptide antigen assembly with MHC protein complex (GO:0002501)	1.43E-02
		synapse pruning (GO:0098883)	1.90E-03
		regulation of microglial cell migration (GO:1904139)	1.89E-03
		regulation of fever generation (GO:0031620)	2.00E-04
		positive regulation of fever generation (GO:0031622)	1.37E-03
		positive regulation of microglial cell migration (GO:1904141)	1.36E-03
		peptide antigen assembly with MHC class II protein complex (GO:0002503)	6.77E-03
		MHC class II protein complex assembly (GO:0002399)	6.75E-03
		leukotriene production involved in inflammatory response (GO:0002540)	4.98E-02
		arachidonic acid metabolite production involved in inflammatory response (GO:0002538)	4.97E-02
		regulation of interleukin-3 production (GO:0032672)	4.96E-02
		positive regulation of leukocyte adhesion to arterial endothelial cell (GO:1904999)	4.96E-02
		positive regulation of cellular response to macrophage colony-stimulating factor stimulus (GO:1903974)	4.95E-02
		positive regulation of response to macrophage colony-stimulating factor (GO:1903971)	4.94E-02
		negative regulation of monocyte activation (GO:0150102)	4.93E-02
		negative regulation of polynucleotide adenylyltransferase activity (GO:1904246)	4.92E-02
		detection of fungus (GO:0016046)	4.91E-02
		negative regulation of autophagic cell death (GO:1904093)	4.90E-02

Supplemental Table 4. Complete list of number of occurrences of each gene on the edges of the network diagram depicting upregulated KEGG pathways in hippocampal tissue from epileptic patients compared to controls.

Gene	Occurrences	Gene	Occurrences	Gene	Occurrences
TNF	56	MMP3	5	BTG2	1
NFKBIA	41	HTR2A	5	LAPTM5	1
IL1B	36	EGR2	4	RPL23	1
FOS	35	SGK1	4	PLCH1	1
MYC	32	MCL1	4	CH25H	1
HLA-DRB5	24	CLDN10	4	MIR222	1
HLA-DRB1	24	RARB	4	DHRS9	1
TLR4	24	GLI1	4	SEC24D	1
HLA-DRA	24	SLC17A6	4	AP3B1	1
HLA-DQA2	24	SOD2	4	SEMA3A	1
HLA-DMB	24	BCL2A1	4	STEAP1	1
GADD45B	23	IL33	4	CSGALNACT1	1
GADD45G	22	CSF2RA	4	B3GNT5	1
PTGS2	19	HTR1D	4	ABCA6	1
GNB4	18	TLR7	3	SLC44A3	1
TLR2	18	PAH	3	NCKAP1L	1
SYK	15	LIFR	3	IRAK3	1
C3	13	CLEC7A	3	GCNT4	1
SOCS3	13	CPB2	3	AQP9	1
CCL2	13	SCIN	3	RORB	1
THBS1	12	OSM	3	SEMA3D	1
CD86	12	HRH1	3	EPHA3	1
CXCL1	11	FPR1	3	PLA2G7	1
HK2	11	ADORA3	3	ALOX5AP	1
IL18	11	WEE1	2	P2RY12	1
BDKRB2	10	HSD17B6	2	SV2C	1
CSF1R	10	CFD	2	MCHR2	1
F2R	10	CX3CR1	2	GLP2R	1
DRD1	10	DOCK2	2	NMU	1
FCGR1A	10	OLR1	2	TAC3	1
CASP1	9	AQP4	2	IRF8	1
ADRB2	8	STMN1	2	VSIG4	1
CXCL3	8	HTR3B	2	CD33	1
CYBB	8	FCAR	2	LILRB4	1

IL6ST	7	RXFP1	2	NR4A3	1
CCL3	7	APBB1IP	2	TREM2	1
MEF2C	7	PRKD3	2	NFKBIZ	1
EGR1	6	CFB	2	KDM6A	1
EDNRB	6	PLD4	2	S100A9	1
KCNQ1	6	ST6GAL1	2	PYDC2	1
C1QB	6	ST6GAL2	2	GBP1	1
SELE	6	SPTLC3	2	TNFRSF10D	1
EIF4E	6	SLC1A3	2	IL1RN	1
C1QA	6	GRIK3	2	TNFSF18	1
NPY	5	IL25	2	CYP4F2	1
NCF2	5	CHRNA3	2	CYP4F3	1
ALOX5	5	S1PR3	2	PPM1B	1
FOSB	5	PPBP	2	SUCNR1	1
HPGDS	5	SPINT1	2	MSR1	1
TNC	5	TTN	2	ZFP37	1
EFNA5	5	IRAK2	2	DPP4	1
NR4A1	5	ZFP36	2	SLC7A7	1
LPAR5	5	CCL8	2	SLC7A11	1
TNFRSF11A	5	EPB41L4B	1	OR2L13	1
